# Variant O89 O-Antigen of *E. coli* Is Associated With Group 1 Capsule Loci and Multidrug Resistance

**DOI:** 10.3389/fmicb.2018.02026

**Published:** 2018-08-31

**Authors:** Susan Harris, Marta J. Piotrowska, Robert J. Goldstone, Ruby Qi, Geoffrey Foster, Ulrich Dobrindt, Jean-Yves Madec, Charlotte Valat, Francesco V. Rao, David G. E. Smith

**Affiliations:** ^1^Institute of Biological Chemistry, Biophysics and Bioengineering, Heriot-Watt University, Edinburgh, United Kingdom; ^2^The Francis Crick Institute, London, United Kingdom; ^3^Veterinary Services, SAC Consulting, Scotland’s Rural College, Inverness, United Kingdom; ^4^Institute of Hygiene, University of Münster, Münster, Germany; ^5^Unité Antibiorésistances et Virulences Bactériennes, Anses Laboratoire de Lyon, Université Lyon-1, Lyon, France; ^6^DC Biosciences Ltd., Dundee, United Kingdom

**Keywords:** *Escherichia coli*, novel O-antigen, lipopolysaccharide, group 1 capsule, multidrug resistance (MDR), quantitative proteomics

## Abstract

Bacterial surface polysaccharides play significant roles in fitness and virulence. In Gram-negative bacteria such as *Escherichia coli*, major surface polysaccharides are lipopolysaccharide (LPS) and capsule, representing O- and K-antigens, respectively. There are multiple combinations of O:K types, many of which are well-characterized and can be related to ecotype or pathotype. In this investigation, we have identified a novel O:K permutation resulting through a process of major genome reorganization in a clade of *E. coli*. A multidrug-resistant, extended-spectrum β-lactamase (ESBL)-producing strain – *E. coli* 26561 – represented a prototype of strains combining a locus variant of O89 and group 1 capsular polysaccharide. Specifically, the variant O89 locus in this strain was truncated at *gnd*, flanked by insertion sequences and located between *nfsB* and *ybdK* and we apply the term O89m for this variant. The prototype lacked colanic acid and O-antigen loci between *yegH* and *hisI* with this tandem polysaccharide locus being replaced with a group 1 capsule (G1C) which, rather than being a recognized *E. coli* capsule type, this locus matched to *Klebsiella* K10 capsule type. A genomic survey identified more than 200 *E. coli* strains which possessed the O89m locus variant with one of a variety of G1C types. Isolates from our collection with the combination of O89m and G1C all displayed a mucoid phenotype and *E. coli* 26561 was unusual in exhibiting a mucoviscous phenotype more recognized as a characteristic among *Klebsiella* strains. Despite the locus truncation and novel location, all O89m:G1C strains examined showed a ladder pattern typifying smooth LPS and also showed high molecular weight, alcian blue-staining polysaccharide in cellular and/or extra-cellular fractions. Expression of both O-antigen and capsule biosynthesis loci were confirmed in prototype strain 26561 through quantitative proteome analysis. Further *in silico* exploration of more than 200 *E. coli* strains possessing the O89m:G1C combination identified a very high prevalence of multidrug resistance (MDR) – 85% possessed resistance to three or more antibiotic classes and a high proportion (58%) of these carried ESBL and/or carbapenemase. The increasing isolation of O89m:G1C isolates from extra-intestinal infection sites suggests that these represents an emergent clade of invasive, MDR *E. coli*.

## Introduction

*Escherichia coli* is a much-studied bacterial species yet remains incompletely understood. This is exemplified by *E. coli*’s “open" genome such that – despite greater than 10,000 genome sequences (complete and incomplete) being readily accessible – sequencing of any further isolates can be predicted to add new content to the pan-genome (Rasko et al., 2008). Additionally, this extent of genome sequence resource is leading to identification of novel genomic features as well as providing for systematic characterization of variable determinants such as surface polysaccharides. These structures include both lipopolysaccharide (LPS) and capsular polysaccharide (CPS) which perform vital functions for bacteria both during colonization of hosts and in the environment outside hosts. Within the host, LPS O-antigen (also termed O-polysaccharide) and CPS contribute to resistance to antibacterial defenses such as complement and antimicrobial peptides as well as resistance to phagocytosis (Miajlovic and Smith, 2014; Abreu and Barbosa, 2017). Some CPS types show strong fitness benefits, a notable example being the *E. coli* K1 capsule which is frequent among extra-intestinal *E. coli* (ExPEC). Parallels are seen in *Klebsiella* for which CPS is a particularly important virulence determinant (Clegg and Murphy, 2016; Paczosa and Mecsas, 2016). Hence, LPS and CPS are described variously as fitness or virulence factors.

At present, there are approximately 180 O-types, 80 K-types, and 50 H-types recognized among *E. coli*, relating to O-antigen, capsule and flagella, respectively. Serotypes are non-randomly distributed among *E. coli* with some showing very strong associations with genotypes and pathotypes. Notable exemplars include serotype O157:H7 which is the archetypal enterohemorrhagic *E. coli*; serotype O104:H4 which are major enteroaggregative strains (EAEC); and serotype O25b:H4 (ST131) which has emerged as a pandemic ExPEC lineage.

Developments of webtools such as SerotypeFinder for *in silico* O:H typing of *E. coli* (Joensen et al., 2015) and Kaptive for *in silico* capsule typing of *Klebsiella* (Wyres et al., 2016) allied to inter-relatedness of serotype with pathotypic characteristics ensure that the O:K:H system remains an informative classification scheme for bacteria such as *E. coli*.

*E. coli* may produce any of four classes of CPS termed groups 1–4 (Whitfield, 2006) plus colanic acid. Group 2 or 3 capsules (G2C or G3C) are the commonest types and strains with these may also co-express with colanic acid; G4C (known as O-antigen capsule) can also be co-expressed with colanic acid and are widely distributed although they may be cryptic; group 1 capsule (G1C) and colanic acid are mutually exclusive, distinct systems. Highly mucoid phenotype may result from substantial production of CPS or colanic acid and excessive production of one or more surface polysaccharides can result in a phenotype variously described as hypermucoid, mucoviscous, or hypermucoviscous. Although these characteristics are more typically identified in *Klebsiella* spp., some exceptional strains of *E. coli* have been described, usually associated with particular mutations (Jayaratne et al., 1993; Potrykus and Wegrzyn, 2004; Zhang et al., 2008; Ionescu and Belkin, 2009; Van Tyne et al., 2016).

Given the significance of LPS and CPS as major surface structures in *E. coli*, *Klebsiella*, and other Gram-negative bacteria, it is unsurprising that they have been the subject of much investigation. Nonetheless, our investigations of multidrug resistant (MDR), ESBL-producing *E. coli* have identified a novel organization in the O-antigen locus. This arrangement is invariably combined with other genomic re-organizations which incorporate carriage of G1C loci. Proteomics and direct examination of cellular polysaccharides confirmed expression of both O-antigen and G1C. Survey of genome sequences and associated metadata show that *E. coli* strains with combinations of this novel O-antigen locus and G1C are geographically widespread and carry a high number of antibiotic resistances including ESBL, carbapenemases and MCR (colistin) resistances. Many recent isolates of this type have been from extra-intestinal samples, suggesting that this may be an emergent MDR clade to which we must be alert. This investigation adds further to our understanding of the adaptability of *E. coli* and the need to remain vigilant to continued evidence of novel characteristics of this deeply studied yet seemingly continuingly variable bacterium.

## Results

### *E. coli* 26561 Exhibits a Mucoid and Viscous Phenotype

**FIGURE 1:**
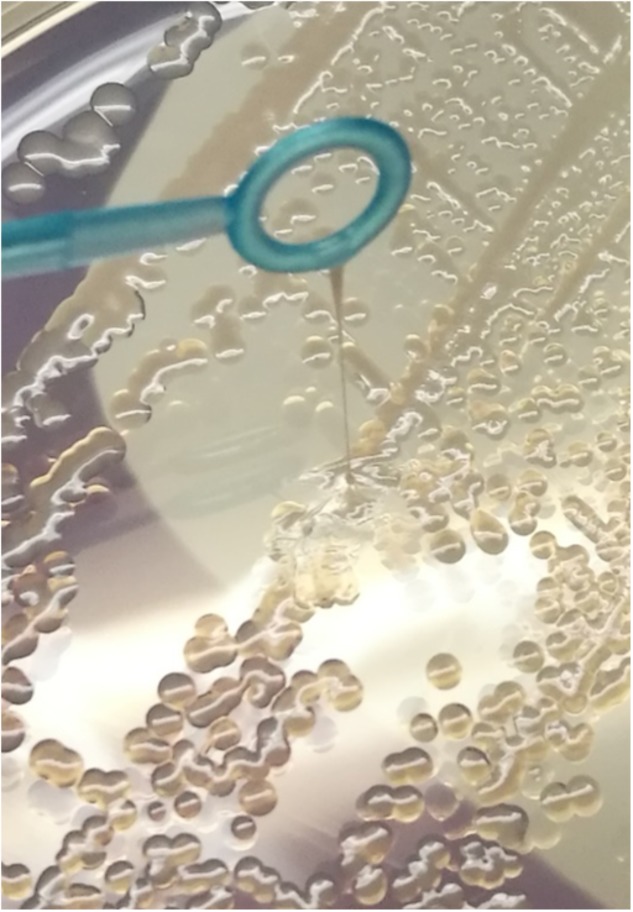
*Escherichia coli* strain 26561 displays a strong “string” effect representative of hypermucoviscosity. This phenotype was evident on all agar media on which this strain was grown and this is a representative image from growth on LB agar.

*Escherichia coli* strain 26561 has been described previously as an ESBL-producing, MDR isolate (Dahmen et al., 2013). Culture of 26561 on a range of agar media revealed a colony characteristic that resembled the “hypermucoviscous” (HMV) phenotype displayed by certain *Klebsiella pneumoniae* strains (Fang et al., 2004; Lee et al., 2006) which presents as a mucoid “string” of ≥5 mm adherent to a microbiological loop. An example of this characteristic is shown in **Figure 1**. A panel of reference strains (including MG1655, NCTC12241, NCTC9089, NCTC11107) did not exhibit either mucoidy or viscosity although strains with the same combination of O-antigen and CPS class as 26561 (see following sections) all displayed mucoidy.

During routine handling of 26561 it was evident that it was not possible to pellet the cells efficiently even under centrifugation conditions of 10 min at 15,000 ×*g* suggestive of production of highly viscous extracellular polymer. A simple assay (Lai et al., 2003; Cheng et al., 2010) was then adopted for relative quantitation of viscosity. A panel of liquid media was assessed and a similar profile of media-dependent relative viscosity was evident. In all liquid media, non-mucoid strains as well as mucoid but non-mucoviscous *E. coli* strains gave negligible relative viscosity values (as defined in “Materials and Methods” section). For *E. coli* 26561, viscosity assays showed three main groupings of medium-dependent relative viscosity: LOW values were obtained during growth in LB, TSB, MHB, and NB; MEDIUM values were obtained by culture in PCB and DMEM-S; HIGH values through culture in M9 minimal medium (with glycerol or glucose as carbon source), MacConkey broth or DMEM-T. Frequently, relative viscosity in these media approached a value of 1, i.e., centrifugation failed to detectably pellet bacterial cells.

Influence of glucose availability on viscosity was further explored in PCB and M9 media: although glucose concentration affected bacterial yield, there was no dose-dependent effect on relative viscosity. Several other parameters were assessed for their effect on the mucoviscous phenotype of 26561: anaerobiosis, growth temperature (37°C, 24°C, and c. 18°C), iron or glucose availability and growth phase showed no discernable influence on mucoviscous characteristics as monitored by string test or viscosity assay (data not shown). Treatment with DNAse also had no effect on viscosity of 26561. A panel of other *E. coli* (including several mucoid strains) lacked viscous phenotype under any condition examined, hence *E. coli* 26561 was unique among this panel in possessing mucoid and viscous characteristics.

### Genomic Characterization and *in silico* Typing

Mucoidy and mucoviscosity are typically conferred by expression of extracellular polysaccharide materials. To identify and characterize potential polysaccharide loci, genome sequencing was carried out on both short-read and long-read platforms as described in “Materials and Methods” section. From a hybrid assembly, three contigs were obtained, the largest of which was 4.72 Mb in length with 27× coverage. The two smaller contigs were 84,087 bases and 50,597 bases long, with coverages of 43× and 64×, respectively. Each of these were resolved into circular contigs by use of Circlator software (Hunt et al., 2015).

The assembled genome was confirmed as *E. coli* through sequence comparison (BLAST) of the entire chromosome sequence. Assembled contigs were submitted to the PlasmidFinder1.3 database (Carattoli et al., 2014) which returned matches (>98%ID) for plasmid replicons FIA, FIB, and IncQ1 to the 84,087 base contig and a 100% match for IncFII to the 50,597 base contig. Submitting contigs to the ResFinder tool (Zankari et al., 2012) which identified *blaCTX*-M-14 on the larger plasmid and *blaTEM*-1, *aph*(3″)-Ib/*strA*, *aph*(6)-Id/*strB*, *aph*(3′)-Ia, *sul2*, and *tetA*(*B*) were carried on the smaller plasmid. Assembled sequence indicated that *catA1* was chromosomally encoded and that this isolate is also resistant to quinolones (nalidixic acid, enrofloxacin and ofloxacin) via substitutions in GyrA (S83L and D87N) and ParC (S80I). This *in silico* characterization of resistance genotype corresponded with previously reportedly resistance phenotype (Dahmen et al., 2013) and specifies 26561 as a multidrug-resistant (MDR) isolate as defined by Magiorakos et al. (2012).

### Surface Polysaccharide Loci

Of the multiple surface polysaccharide structures potentially carried by *E. coli*, we found loci for LPS, enterobacterial common antigen (ECA), cellulose (pseudogenized), poly-*N*-acetyl-glucosamine (PNAG) and two distinct capsule systems.

#### Capsular Polysaccharide

The known capsule types of *E. coli* are groups 1–4 and colanic acid (Corbett and Roberts, 2008; Whitfield, 2009) of which the most widely distributed is the latter. In the 26561 genome, we did not identify colanic acid, group 2 or group 3 CPS loci although a match was found to group 4 capsule (G4C – *gfcABCDE-etp-etk*). Notably in 26561 this G4C locus appears to be intact and lacks the interrupting insertion sequence present in the MG1655 genes (Peleg et al., 2005).

**FIGURE 2:**
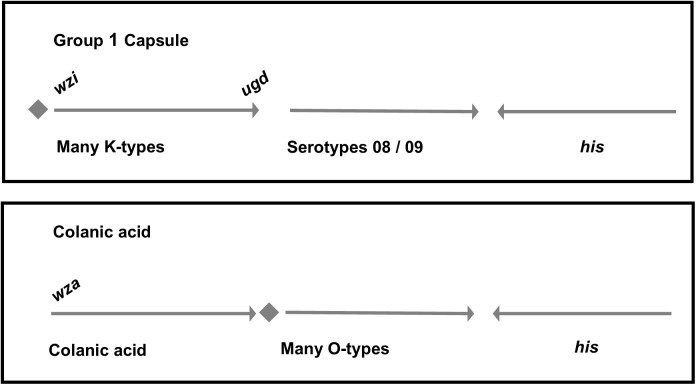
Differences in the genetic organization between *E. coli* group 1 and colanic acid capsules. In both groups, capsule and O-antigen gene clusters are located near the operon for histidine biosynthesis. In group 1 capsules, *galF* and a JUMPStart sequence (represented together as a diamond shape) are located upstream of the capsule genes. The first structural gene of the capsule, *wzi*, is unique to group 1 capsules. By comparison, strains which produce colanic acid have the *galF* and JUMPStart region upstream of the O-antigen. The final gene in the colanic acid locus is *wcaM* which is absent in group 1 capsule loci.

In *E. coli*, the colanic acid locus is located in the genomic region between *yegH* and *hisI* together with the gene cluster which confers O-antigen biosynthesis (presented schematically in **Figure 2**). In the 26561 genome sequence, the region between *yegH* and *hisI* comprises twenty genes with functions consistent with polysaccharide biosynthesis and transport, in addition to hypothetical gene products and insertion sequence elements (summary annotation is provided in **Supplementary Table 1A**). Neither colanic acid nor O-antigen biosynthetic loci were present, rather, this region featured genes typical of G1Cs, specifically *galF*-*hypothetical* (PAP2) upstream of *wzi-wza-wzb-wzc-wbaP*. Thus, colanic acid and adjacent O-antigen loci are replaced by G1C, a scenario which is distinct from that previously reported for other G1C-containing *E. coli* (Whitfield, 2009) in which G1C replaces colanic acid adjacent to O-antigen locus.

**FIGURE 3:**
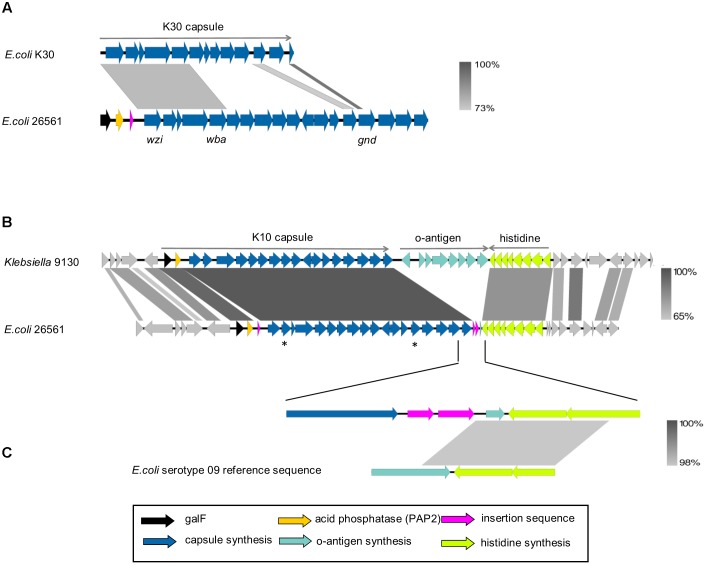
Alignment of group 1 capsule loci. **(A)** Prototypical *E. coli* group 1 capsule locus (ec_K30) aligned to capsule locus of strain 26561. The reference sequence for *E. coli* K30 capsule (accession AF104912.3) spans 16,109 nucleotides comprising genes from *wzi* to the 5′ end of *gnd*. The alignment shown corresponds to nucleotide identity of 73% between the first five genes; *wzi-wza-wzb-wzc-wbaP*, which are common to group 1 capsules. **(B)** *Klebsiella* NCTC 9130 is the reference strain for the *Klebsiella* K10 serotype. Shown is its nucleotide alignment to *E. coli* 26561 by BLAST with percent identities of the alignments represented by the gradient shown on the right. The central region of the capsule locus is the most strongly conserved, with genes between *wza* and *wbaZ* (marked by asterisks in the diagram) each sharing 99–100% identity. In 26561, an insertion sequence is present upstream of the first structural gene of the capsule, *wzi*, and two are found after the last gene in the locus*, ugd*. **(C)** The upper row is a close-up of 26561, where the insertion sequences are present following *ugd*. A small coding sequence of 54 amino acids is present downstream of the insertion sequences and upstream of the *his* operon. This is found to match 100% to the final 162 nucleotides of the *E. coli* O8 and O9 serotype reference sequences (bottom row; only O9 shown).

The prototypical G1C locus from *E. coli* strain E69 (serotype O9a:K30:H12; accession AF104912.3) has been extensively characterized over the past 30 years. This capsule locus sequence spans approximately 16,000 bp from *wzi* to *gnd* and alignment of G1C loci from E69 and 26561 is presented in **Figure 3**. This comparison demonstrates conservation in assembly, transport, membrane-anchoring and biosynthetic components (*wzi*, *wza*, *wzb*, *wzc*, *wbaP*) and in the non-coding upstream region. The divergence in sequences of glycosyl-transferases and other genes encoded in the central region is indicative that the G1C type (K-antigen) of 26561 is distinct from K30.

The genomic region of 26561 from *galF* to *ugd* (∼24,500 bp) was used to search NCBI nucleotide collection and whole genome shotgun contigs (WGS) databases (last searched November 2017) resulting in no extensive matches to *E. coli* (40% coverage at most) yet identifying four matches to *Klebsiella* with >90% identity over >90% of sequence. Hence, this CPS locus is rare in *E. coli* and appears to have been a relatively recent horizontal acquisition of capsule from *Klebsiella*. The matching *Klebsiella* capsule typed as K10 and the extent of identity between the 26561 locus is presented in **Figure 3**. The terminology KP_K10 will be used to distinguish from *E. coli* K-types.

#### O-Antigen Locus

Submission of the complete 26561 nucleotide sequence to the SerotypeFinder database returned a result of serotype O89 by matching of the *wzt* and *wzm* genes by 93.49 and 94.10% identity, respectively. In the region encompassing *galF-gnd*, the O89 locus is highly similar to O101 and O162 loci (DebRoy et al., 2016: Iguchi et al., 2015) and the *wzm* and *wzt* sequences of 26561 show 92.6% and 93.4% identity, respectively, to those of both O101 and O162. Locating the chromosomal position enabled comparison of the O-antigen genomic region of 26561 to those in the reference sequence for the O89-serotype locus. From an initial inspection of the 26561 locus in Artemis, the first nine genes (from *galF*) conformed to the published composition of the O89 locus, although we noted that the 26561 sequence retained a partial *wcaM* (the final gene of the colanic acid locus) and the *gnd* gene was severely truncated (henceforth *gnd#*), both being disrupted by insertion sequences. To examine differences further, the whole genome reference sequence for O89 strains (strain NCTC9089; European Nucleotide Archive SAMEA3403041) was aligned with the corresponding region in 26561.

**FIGURE 4:**
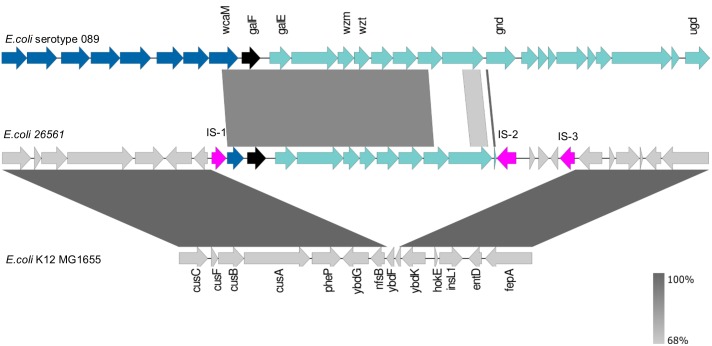
Reference strain for *E. coli* serotype O89 (NCTC9089) aligned to O-antigen genes in *E. coli* 26561. The reference sequence for *E. coli* serotype O89 is shown in the upper track. The black arrow marks *galF* and the start of the O-antigen locus with its remaining genes to *ugd* shown in green. The dark blue arrows preceding *galF* represent the colanic acid locus. The *wzm* and *wzt* genes which are used for *in silico* serotyping by SerotypeFinder are labeled. Their nucleotides are 94.10% and 93.49% identical to the corresponding genes in *E. coli* 26561 (middle track). In *E. coli* 26561 we see that a partial *wcaM* (the final gene in the colanic acid locus) is present but interrupted by an insertion sequence at its 5′ end (pink arrow: IS-1). The furthermost match is to the beginning of the *gnd* gene. The protein coding sequences for the two genes preceding *gnd*, a methyl transferase and glycosyl transferase, are 47% and 42% identical, respectively, but are a 100% match to other proteins with the same function in the NCBI database. The genes which flank the insertion sequences marked IS-1 and IS-3 in *E. coli* 26561 are *nfsB* and *ybdK*. The bottom sequence illustrates that these genes are usually separated by two small hypothetical genes, *ybdF* and *ybdJ*, in *E. coli* K12_MG1655 (*ybdJ* is not labeled in the diagram).

From the alignment (**Figure 4** and further elaborated in **Supplementary Presentation 1**) it became clear that there were additional differences between these loci. Specifically, the two genes preceding *gnd#*, annotated as a methyl-transferase and a glycosyl-transferase, share only 47% and 42% amino acid identity to their counterparts in NCTC9089 whilst BLAST searches with the 26561 sequences show 100% matches to other proteins with the same function in the NCBI database. Closer inspection of the protein annotated as Gnd# in 26561, indicated that it is only 33 amino acids long, compared to 468 residues in the reference. In addition to the premature stop codon, there is also a divergence in the coding sequence: the first 22/33 amino acids are a 100% match, but the remaining 10 amino acids – RALLNKSDFG – have no match in the reference genome (**Supplementary Table 1B** lists the genes from *galF* to *gnd*, their corresponding annotations and identity in 26561). Of further note is a region downstream of *gnd#* in 26561 which comprises two insertion sequences flanking three short coding sequences which are annotated as putative Nlp-type regulator, GNAT family *N*-acetyl transferase and a hypothetical protein.

Divergence is also evident in the genes flanking the locus in 26561 and the reference O89 genome. In the reference the adjacent loci are colanic acid (*wca*) upstream and *his* operon downstream, which is the expected arrangement for the location of the O-antigen. In 26561 the insertion sequences that bracket the locus are preceded by *nfsB* upstream and followed by *ybdK* downstream. The genes upstream of *nfsB* include those coding for copper/silver export; those downstream of *ybdK* include the iron-acquisition genes conferring enterobactin production. Aligning to the enterobactin-*ybdK* and *nfsB*-copper/silver regions in *E. coli* MG1655 (lower section of **Figure 4**) shows that these encompass *ybdF* and *ybdJ*, two small hypothetical genes which are not present in 26561.

Taking into account (i) the truncation of O-antigen locus (relative to conventional O89), (ii) sequence divergences within remaining genes, (iii) foreshortening of *wcaM* and *gnd*, (iv) juxtaposition of presumptive regulatory locus, and (v) interspersion of insertion sequences, it seems probable that the O-antigen in 26561 is a recent rearrangement into this novel genomic context.

### Expression of Polysaccharide Biosynthetic Proteins

The atypical organization and location of polysaccharide biosynthetic loci led us to conjecture whether LPS and CPS were expressed. Corroboratory evidence was sought in a protein expression dataset from 26561 (to be reported in full separately) and direct analysis of cell-associated polysaccharide components (see following section). As described in “Materials and Methods” section, a Tandem Mass Tag (TMT) mass spectrometry approach was used to assign and quantify protein expression. In matching peptides to the 26561 protein database, we used only those which matched uniquely to a given protein such that paralogs (e.g., two GalF variants) could be distinguished from each other. This proteome analysis detected a total of 53% (2451 of the 4577) potential proteins encoded by 26561 genome, providing coverage similar to the upper range in other LC-MS/MS analyses of unrelated *E. coli* clinical isolates (e.g., Pettersen et al., 2016, 2018; Møller et al., 2017).

**FIGURE 5:**
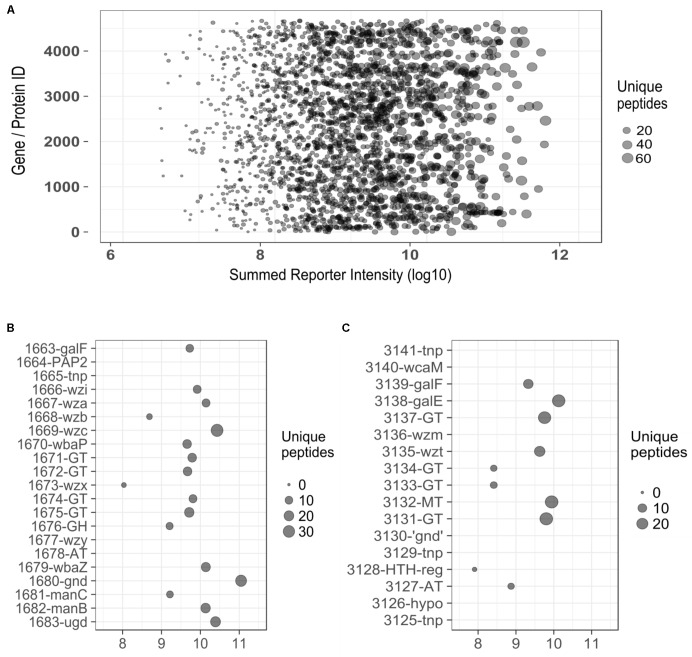
**(A)** Plot displaying the range of reporter intensities for all 2451 proteins identified in *E. coli* 26561. **(B)** Expanded section of plot **(A)** detailing identifications for proteins in the capsule region (*y*-axis). **(C)** Expanded section of plot **(A)** detailing identifications for proteins in the ‘O-antigen’ region (*y*-axis). ‘Summed Reporter Intensity’ (*x*-axis in all figures) represents the total signal detected from 10 TMT-labeled samples. tnp, transposon; GT, glycosyl-transferase; GH, glycosyl-hydrolase; AT, acyl –transferase; MT, methyl-transferase; ‘gnd,’ truncated gnd.

Evidence for expression of both capsular and O-antigen loci from the proteome dataset is convincing; as illustrated in **Figure 5A**, reporter intensities for the data as a whole span ∼5 orders of magnitude (range 3.1 × 10^6^ – 3.6 × 10^11^), with the average value for CPS (**Figure 5B**) and O-antigen (**Figure 5C**) biosynthesis proteins (9.4 × 10^9^) being toward the upper end of this range. Of 38 coding sequences between the two loci, 11 are not detected as proteins. Of these, two are annotated as ‘hypothetical’ proteins (1664 and 3126), four are transposon proteins (1665, 3141, 3129, and 3125) and two are known to be truncated (3140 – *wcaM* and 3130 – *gnd*). Of the three that remain, (1667 – *wzy*, 1678 – AT, and 3135 – *wzm*) two are small transmembrane proteins which are known to be difficult to identify as they may be poorly solubilized and digested in the sample preparation stages.

Tandem Mass Tag data were also examined for evidence of expression of G4C (O-antigen capsule) system which comprises seven gene products and has some similarities to G1C. Of the G4C proteins, GfcE, Etp, and Etk (which are paralogous to G1C-encoded Wza, Wzb, and Wzc, respectively) were detected by only 3, 2, and 3 peptides. This observation suggests that G4C makes negligible – if any – contribution to the mucoid phenotype of *E. coli* 26561 despite this locus being intact.

Hence, we can postulate that O-antigen and CPS biosynthetic loci are functional and are presumably responsible for the observed mucoid or mucoviscous phenotype. We further investigated expression of LPS and CPS by several strains.

### Characterization and Distribution of O89 O-Antigen and G1C Loci Among Representative Complete *E. coli* Genomes

An initial BLASTp search (December 2016) of the NCBI nr database with the 33 amino acid Gnd# sequence of 26561 returned only one 100% match with the human intestinal isolate *E. coli* MS-175. This had been assembled by short read data which made it difficult to accurately compare to 26561. A contemporaneous search with the corresponding nucleotide sequence returned five 100% matches (to strains Sanji, H8, Y5, 6409, EC590) which had been sequenced by PacBio to generate complete genomes. Genome sequences were downloaded and submitted to SerotypeFinder: in each case the O-type result returned was identical to that obtained for 26561, i.e., serotype O89 with 94.10% ID to *wzm* and 93.49% ID to *wzt* indicating that the 26561-like O89 locus may be present in these strains. Sequences were annotated using the same program and parameters as used for 26561, and O-antigen regions were located. In each case the genes within the O-antigen and the chromosomal location (between *nfsB* and *ybdK*) were identical to that of 26561. Alignments of these loci from selected strains with 26561 O-antigen locus were performed thus confirming locus content and organization (not shown).

**FIGURE 6:**
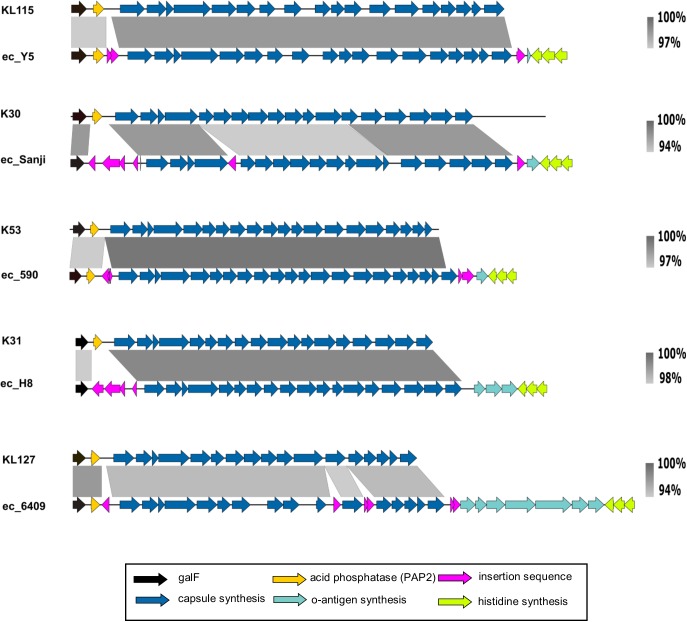
Group 1 capsule loci of representative *E. coli* ‘O89m’ strain capsule loci aligned to matching *Klebsiella* reference capsule sequences. Group 1 capsules of O89m strains display high similarity to those in *Klebsiella*. The nucleotide sequences between the capsule (blue) and histidine (lime green) biosynthetic genes match to the 3′ ends of either O8 or O9 O-antigen loci.

In these completely sequenced strains we also investigated the region around the *his* operon, identified in 26561 as the location of G1C. A G1C-type locus was indicated in each by the presence of *wzi-wza-wzb-wzc-wbaP* downstream of *galF*, however, the remaining genes did not match the KP_K10 capsule of 26561 or the prototype Ec_K30, nor did they match each other. To our knowledge no tool exists for the typing of *E. coli* capsules, however, more has been done to characterize *Klebsiella* capsules genetically. The recently published tool, Kaptive (Wyres et al., 2016) was created to predict *Klebsiella* capsule types using short and long-read WGS data. Submitting our sequences speculatively, we were surprised that in each case a *Klebsiella* capsule type was returned which indicated close matches. We obtained the relevant *Klebsiella* capsule sequences from NCBI and performed alignments with each of the selected complete genome sequences (**Figure 6**). In all selected genomes, the only regions of non-alignment are to insertion sequences in the *E. coli* strains which otherwise display high level of sequence similarity to *Klebsiella* capsular loci.

In each of the six G1 capsule strains for which we performed alignments we noted a variable number (one to seven) of coding sequences between the transposon at the distal end of the capsule locus (following *ugd*) and the *his* operon, in the region where we would normally expect to find the O-antigen gene cluster (**Figure 6**, green arrows). We extracted this region from each sequence and found them to match O8 or O9 serotype loci with high confidence (>99% nucleotide identity as summarized in **Supplementary Table 1C**). In all instances, the initial genes for O8 or O9 loci are absent. Notably, although O8 and O9 loci differ substantially from each other (Iguchi et al., 2015; DebRoy et al., 2016) the final gene – *mtfC* – shares > 94% identity over its entire length and is identical over the distal 150 nucleotides, therefore it is not possible to distinguish between O-types O8 and O9 in which remnants are shorter than this. Retention of variable content of residual O8/O9 locus sequences further identifies the major genome reorganization events in strain 26561 and other strains with the foreshortened O89 locus.

### Characteristics of LPS and CPS Expressed in 26561 and Other O89 *E. coli* Strains

Given the atypical organization of O-antigen and capsular loci, and the novel combination of O and K types in *E. coli* 26561 and other O89m strains for which complete genomes are available, we speculated whether both LPS and CPS were produced by strains of this type. As indicated above, proteomics identified biosynthetic proteins for both loci in prototypic strain 26561. We sought corroboratory evidence by examining cellular and extracellular polysaccharide production by a panel of O89 strains. Specifically, we surveyed genome sequences from our strain library and identified in our collection five additional strains all possessing the same composition of O89m genes as 26561; none of our isolates possessed a conventional O89 locus (summary information for all O89 and O89m strains is provided in **Supplementary Data Sheet 1**). Also examined were reference strains for conventional O89 and O9 O-antigens, respectively, NCTC9089 and NCTC11107.

**FIGURE 7:**
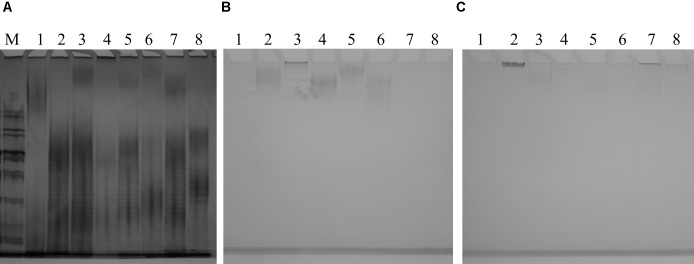
**(A)** Silver-stained gel of lipopolysaccharide preparations from a panel of O89m *E. coli* isolates and reference strains. Strains examined are: NCTC9089 (O89 reference strain) – lane 1; 26561/G246 – lane 2; C720825/G315 – lane 3; C021088_1/G189 – lane 4; C447731/G324 – lane 5; 2308/G76 – lane 6; C232039_2/G310 – lane 7; NCTC11107 (O9 reference strain) – lane 8. With the exception of NCTC9089, strains showed a clear ladder pattern which was almost indistinguishable among O89m strains and distinct from both reference O89 and O9 strains. Alcian blue-stained gels of cell-associated **(B)** and extracellular **(C)** polysaccharides from a panel of O89m *E. coli* isolates and reference strains. Strains are: NCTC9089 (O89 reference strain) – lane 1; 26561/G246 – lane 2; C720825/G315 – lane 3; C021088_1/G189 – lane 4; C447731/G324 – lane 5; 2308/G76 – lane 6; C232039_2/G310 – lane 7; NCTC11107 (O9 reference strain) – lane 8. With the exception of NCTC9089, all other strains possess group 1 capsule loci and demonstrate staining of high molecular weight material in cell-associated and extracellular samples.

O-antigen confers a “smooth” phenotype which is represented by a “ladder” pattern when LPS is separated by polyacrylamide gel electrophoresis (**Figure 7** and **Supplementary Figure 2**). Since the 26561-like O89m lacks several genes present in “intact” O89 locus and since strains with this foreshortened locus possess varying numbers of O8/O9 genes, this may affect LPS ladder pattern. Despite this, 26561 and similar O89m strains all presented ladder patterns typical of smooth (O-antigen-competent) LPS and which was clearly distinct from that of both the O89 and the O9 reference strains. Therefore, despite possessing an O-antigen locus terminating at *gnd*, intact, “smooth” type LPS is produced.

Alcian blue staining of CPSs (**Figures 7B,C**) shows cellular and extracellular polysaccharide preparations from the same panel of strains. Among these only NCTC9089 lacks G1C. Samples underwent exhaustive digestion with DNAse during preparation of polysaccharides, thus the possible contribution of DNA to this feature can be excluded. As anticipated from matches to distinct *Klebsiella* CPS types, profiles of cellular and extra-cellular polysaccharides varied among strains. It is also notable that the two strains carrying KP_K10 capsule loci (26561 and C720825) show different profiles and distribution between cellular and extracellular fractions inferring that strain-specific determinants may contribute to polysaccharide synthesis and surface presentation. Our initial genomic comparison of these KP_K10 strains shows that chromosome content is highly similar although incomplete genome restricts detailed comparisons. Verification of these postulations will be subject to separate investigation.

### Characterization of Clade of O89- and G1C-Positive *E. coli* Strains

Reports on *E. coli* O89 are rare, therefore we conducted a survey (March 2018) of publicly available *E. coli* genome sequences using nucleotide sequences representing entire O-antigen loci from 26561 and NCTC9089 as well as individual coding sequences and protein sequences from these strains. From this survey, a total of 212 *E. coli* complete or WGS genomes were identified (**Supplementary Data Sheet 1**). Surprisingly, among these genomes, only ten (including NCTC9089) possessed a region encompassing all 20 genes from *wcaM* to *ugd* whilst the remainder terminated with a truncated *gnd*. Interestingly, the 26561 region encompassing *galF*-*gnd* also matched at 99% identity over 99% of the sequence to a genome identified as *Citrobacter braakii* strain FDAARGOS_290 (GenBank accession CP022049.1). In all instances matching 26561 sequence, the predicted protein sequence for truncated *gnd* terminates RALLNKSDFG, a motif that is restricted to C-terminal of twenty-two diverse proteins from *E. coli*, *Serratia*, *Enterobacter* and *Klebsiella* (last searched March 2018). O-serotype was cross-validated using the SerotypeFinder tool with the result returned in every case (including the *C. braakii* strain) was identical to that obtained for 26561, i.e., serotype O89 with 94.10% identity to *wzm* and 93.49% identity to *wzt*. In 25 complete *E. coli* genomes matching O89m, upstream and downstream flanking genes to this locus were identified as *nfsB* and *ybdK*.

From the *E. coli* O89 complete genomes examined to this point, it is evident that this serotype determinant frequently associates with G1C CPSs. To determine how consistent this is as a feature of O89/O89m, genomes for these 212 strains were surveyed for *wzi*/Wzi as a surrogate for G1C. Among the 212 O89-positive genomes, a total of 202 were identified which also possessed G1C. Our survey also included a preliminary assessment of general G1C distribution among the entire set of available *E. coli* complete genomes which at the time of sampling (February 2017) identified 197 *wzi*-positive *E. coli* strains comprising O9 (*n* = 63; 32%), O8 (*n* = 31; 16%), untypeable (*n* = 23; 11%); among these, O89 was predominant (*n* = 80; 41%). A more extensive survey of *wzi* distribution among *E. coli* is underway and detection of this gene may be a useful adjunct in surveillance and diagnostic settings. As expected, O89 strains possessing G1C lacked the locus encoding colanic acid biosynthesis and were all of the 26561 O89m type. In contrast, colanic acid-encoding strains all possessed the conventional O89 O-antigen locus (as for NCTC9089).

Further exploration by *in silico* typing of O89 strains identified phylogenetic relatedness based on *in silico* prediction of STs – 157 of 202 O89m strains were identified as members of the ST10 complex (encompassing ST10, ST167, ST617, ST34, ST44, ST209, and ST48; full information provided in **Supplementary Data Sheet 1**). Despite close phylogenetic relatedness, these O89m isolates originate from disparate geographic settings and include commensal/fecal isolates as well as clinical isolates. Where information is available regarding clinical status, 37.5% (63 of 168) were assigned an extra-intestinal origin (most frequently blood, urine, and mastitis with ten further sites of origin).

Since prototype strain 26561 was originally identified as ESBL-positive, multidrug resistant strain, all O89 strains were assessed for carriage of transmissible resistance factors through ResFinder. This revealed an exceptionally high prevalence of accessory antibiotic resistances among O89m strains which was supplemented through substitutions in GyrA, GyrB, ParE, and ParC consistent with quinolone resistance. Greater than 95% of O89m strains showed carriage of one or more accessory resistance determinant compared to approximately 50% of *E. coli* as an entire population (Goldstone and Smith, 2017). Resistances for aminoglycosides, sulphonamides, tetracycline, or β-lactams/cephalosporins were most common, each being detected in >80% of O89m strains. Most (90%) O89m strains can be classified as MDR (three or more resistances) and the mean, median and modal values for resistance classes were 6, 7, and 8, respectively. In contrast, O89 strains showed mean, median and modal values of 1, 1, and 0, respectively, and for *E. coli* as an entire population these values were 1.6, 0, and 0, respectively (Goldstone and Smith, 2017). Among O89m isolates, 118 (58%) carried either extended-spectrum β-lactamases (ESBLs) or carbapenemases of CTX-M, CMY, SHV, NDM, KPC or OXA-48 classes. Sixty-three carried one or more ESBLs, 6 carried carbapenemase, and 49 carried both ESBL and carbapenemase. No O89 strains carried any of these resistances.

## Discussion

*Escherichia coli* is a well-studied bacterial species despite which it remains incompletely understood, partly as a result of its adaptability. This bacterium has adopted a diverse range of lifestyles of which characteristics are conferred through the flexibility in genome content. Diversity in genotype and pathotype is reflected in classification schemes for *E. coli* and the O:K:H system for O-antigen, capsule and flagella typing has remained a mainstay. Systematizing gene content of bacterial polysaccharide-biosynthesis loci presents significant challenges; nonetheless, comprehensive categorization of *E. coli* O-antigen loci (Iguchi et al., 2015; Joensen et al., 2015; DebRoy et al., 2016) has been achieved. For *E. coli*, CPS loci remain to be similarly categorized although the recently described systematization for *Klebsiella* CPSs serves as a model (Wyres et al., 2016). Indeed, some capsule types are common between *Klebsiella* and *E. coli*, e.g., *Klebsiella* K20 (KP_K20) is equivalent to *E. coli* K30 (Ec_K30) and our study indicated that exchange of this locus may extend further. These developments in *in silico* serotyping ensure that the O:K:H system still represents an informative classification scheme for *E. coli*.

### O89-Encoding Genomic Loci Display Two Distinct Variants Resulting From Genomic Rearrangements

To date, *E. coli* of serotype O89 have been rarely reported (Füst et al., 1977; Elekes et al., 1988; Hibberd et al., 1991; Yamamoto et al., 1996; García-Aljaro et al., 2005; Dutta et al., 2011; Ahmadi et al., 2012; Garza-Ramos et al., 2018), however, our survey has identified more than 200 O89 strains among sequenced genomes (complete and WGS). With *E. coli* 26561 as prototype, inspection of the O89 locus and comparison with that of the locus from reference strain NCTC9089 identified several notable distinguishing features. Most evident were truncations of the O-antigen locus in *wcaM* and *gnd* at either end of the locus both of which were immediately adjacent to insertion element sequences, indicative of genomic reorganization. The truncation in *gnd* results in a predicted protein of 33 amino acids (in contrast to 468 residues of wild-type Gnd), thus it can be predicted that this gene is non-functional. Comparison of these truncated sequences with O89 reference sequence from NCTC9089 showed ten additional genes were present in reference O89 sequence distal to *gnd* and ending with *ugd*. These encoded further polysaccharide biosynthesis genes typical of O-antigen loci. Inspection of over 212 other O89 strains showed that this O-antigen locus truncation was common to the majority (>95%) of these strains with only 10 of 212 retaining conventional O89 gene content – we use the term “O89m” for these truncated variants. This assessment also revealed remnants of O-antigen biosynthetic genes matching to either O9 or O8 types adjacent to *his* locus, a site described as the location of O-antigen loci in *E. coli* and confirmed in 100% of 573 *Klebsiella* strains recently surveyed (Follador et al., 2016). In strains carrying the conventional O89 type, the O-antigen locus was in the expected sitting immediately downstream of colanic acid (which is preceded by *yegH*) and upstream of *his*, whereas the location of the O89m locus was between *nfsB* and *ybdK* genes. Although the O89m locus mapped between *nfsB* and *ybdK* in the majority of cases, there was a small minority of instances in which this was not the case – these genomes are identified in **Supplementary Table 1D**. In five of the strains (3c112, G188, G229, G233, G315), the *ybdK-ybdJ-ydbF-nfsB* region was uninterrupted, indicating a different site for the O89m locus. However, investigation of these assemblies, which were highly fragmented, showed the O89m genes to form short contigs bounded by insertion sequences and so we were unable to link them to a location. A high quality assembly (composed of only five contigs) is available for the sixth of these strains, B41, in which the location of the O89m locus was identified between *tomB* and *dtpD*. Interestingly, of the of the *ybdK-nfsB* genes only *ybdK* was found in this strain, indicating further genomic re-arrangements in B41. To our knowledge, these are only the second reported instances of O-antigen localization in *E. coli* at novel genomic sites as was recently noted for O62 (Hou et al., 2017). Taken together with the flanking of this O89m O-antigen region with insertion sequences, this is indicative of a major genomic reorganization event.

### The More Prevalent O89 Variant Is Associated With Carriage of Group 1 Capsule (G1C)

Further survey of genomes of O89m strains identified additional polysaccharide loci including one encoding intact Gnd. Interrogation of this locus identified it as encoding a G1C which was distinct from the prototypic K30 G1C. Consistent with previous reports (Amor and Whitfield, 1997), our initial survey indicated that G1C-positive strains were commonly O9 (32%) or O8 (16%) and a further 11% of strains comprised other O-types or for which O-type could not be categorized. Significantly, the most frequent O-type associated with G1C was O89m which represented 41% of all G1C-positive *E. coli* strains.

Searching the sequence databases with the nucleotide sequence of the 26561 G1C locus matched with *Klebsiella* capsule type KP_K10. Intriguingly, many of the O89m genomes matched with high confidence to *Klebsiella* capsule types with various combinations of insertion sequences bracketing *wzi* to *ugd*. Among O89m strains, the G1C gene cluster (regardless of K-type) was located between *yegH* and *his* genes, the region which is normally considered the location of colanic acid together with O-antigen genes or G1C together with O-antigen genes (as exemplified by *E. coli* O9a:K30:H12 strain E69). Taking into consideration this group of approximately 200 O89m *E. coli* strains, it is evident that major genomic reorganization concurrent with genome island acquisition has taken place among these strains. These events have involved replacement of colanic acid and adjacent O-antigen loci between *yegH* and *his* genes with G1C, an event that has occurred on multiple occasions, introducing multiple capsule types with close identity to those of *Klebsiella* spp. G1C acquisition was presumably preceded by acquisition of the O89m locus – most frequently by insertion between *nfsB* and *ybdK* which itself appears to be a recent reorganization given the restricted distribution of these events among *E. coli*.

### O-Antigen and Capsular Polysaccharides Are Expressed Despite Genomic Rearrangements

Consequent to rearrangements in O-antigen and CPS loci are truncation of *gnd* (6-phosphogluconate dehydrogenase, decarboxylating) in the O-antigen locus and its presence in intact form in the G1C locus. Although Gnd is non-essential for *E. coli* viability, this enzyme plays a significant role in metabolism (pentose phosphate pathway) which has knock-on roles in fitness, notably as a substrate for several biosynthetic pathways including polysaccharides (Jiao et al., 2003; Zhao et al., 2004). Genome re-organizations have also resulted in acquisition of two copies of *galF* (encoding a regulatory subunit for GalU, glucose-1-phosphate uridylyltransferase); this redundancy and divergence of *galF* is common across the G1C-carrying strains examined. Taking strain 26561 as exemplar, the two copies of *galF* show 77% identity at the nucleotide level and the corresponding proteins are approximately 95% identical. Compared to GalF of *E. coli* MG1655, the G1C-associated GalF and O-antigen-associated GalF show 91% (273/299) and 95% (284/299) identity in amino acid sequences, respectively. GalF has been shown to interact with and modulate the function of GalU, glucose-1-phosphate uridylyltransferase. Like Gnd, this enzyme performs core metabolic roles and contributes to biosynthesis of surface polysaccharides (Marolda and Valvano, 1996).

Given the novel combination of surface polysaccharides O89m and KP_K10, the truncation and rearrangements between loci and the unusual juxtaposition of these within core genome, it was important to confirm production of both O-antigen and capsule. Proteomics confidently detected in *E. coli* 26561 the majority of proteins involved in CPS and O-antigen biosynthesis and export and also as well as lipid-A and core biosynthetic proteins of LPS. Gnd and both copies of GalF are expressed despite the alterations affecting *gnd* and *galF*. The intact Gnd protein is the most strongly expressed protein among all detected O-antigen and capsule polysaccharide biosynthetic proteins. Expression of both copies of GalF is of note although it is not apparent whether they play redundant, complementary of competitive roles in metabolism and polysaccharide synthesis.

Corresponding with protein expression, there appears to be no evidence for deficit in surface polysaccharide expression or composition. Visualization of LPS (**Figure 7**) from six O89m strains show that they all exhibited a staining pattern typical of “smooth” LPS with an obvious “ladder” of O-antigen repeat units. All six O89m strains examined possess a G1C locus and all displayed high molecular weight alcian blue-staining polysaccharide in cell-associated, extracellular or both fractions. Pattern varied among strains, consistent with the carriage of distinct capsule types among these strains. Among these, 26561 was notable in production of a dense band of very high molecular weight material in the extracellular fraction with similar material just discernible in the cell-associated fraction. It is presumed that this predominantly extracellular polysaccharide is responsible for the profound mucoviscosity of 26561.

Proteomics also provided an opportunity to assess which regulatory proteins associated with polysaccharide expression were detected. LPS (lipid-A, core, and O-antigen) is an essential surface structure and its expression is constitutive and as noted above, G1C is reported as constitutively expressed. Expression of both LPS and CPS loci may be subject to additional regulatory control via alternative sigma factors, multiple signaling relays and other regulators in response to growth status, envelope stress and other cues. Important among these regulatory processes are the Rcs regulatory cascade (Whitfield and Roberts, 1999) and RfaH (Bailey et al., 1997), which is a transcriptional anti-terminator which recognizes JUMPstart/*ops* sites and is reported to play important roles in expression of long operons, particularly surface or secreted structures including both LPS and CPS expression. Among G1C-positive strains, both O-antigen and capsule loci possess JUMPstart sequences sited, respectively, between *galF* and *galE* and between *galF* and *wzi*, consistent with the participation of RfaH-JUMPstart in expression. Rcs is a multicomponent regulator comprising core components RcsB, RcsC, RcsD, and RcsF which were all detected in the proteome as was RfaH. The proteins annotated as Nlp regulator and acetyltransferase (sited along with a hypothetical protein are located adjacent to O-antigen locus and are flanked by insertion sequences in O89m genomes) were detected with only low confidence and their expression as well as any functional contribution will require independent investigation. Acetyltransferases (ATase) integral to the *Klebsiella* K1 locus have been shown to contribute to gene expression (Ho et al., 2011), although as yet, roles of neither the Nlp regulator nor ATase have been confirmed. We speculate that these gene products participate in regulation of polysaccharide and/or other gene expression.

Determinants regulating expression of O89m and G1C and the extent of any potential regulatory interplay between these polysaccharide loci have yet to be defined. Similarly, determinants of the hypermucoviscosity of 26561 remain to be specified and characterized experimentally. It will be particularly significant to compare with other *E. coli* O89m strains encoding KP_K10 capsule as well as characterizing among wild-type *E. coli* isolates showing hypermucoviscosity to provide comparison with *Klebsiella*. Further comparative and functional genomics investigations will be required for verification of these overlapping hypotheses.

### Strains Possessing O89 and G1C Display Mucoid or Hypermucoviscous Phenotype

Carriage of capsule typically confers a mucoid characteristic upon strains and all G1C strains examined produced mucoid colonies on several different agar media. Among the G1C strains, 26561 was distinct in exhibiting a mucoviscous phenotype. On agar media strain 26561 was profoundly “string test” positive and in liquid media 26561 proved strongly positive in a viscosity assay. These characteristics are consistent with “hypermucoviscous” phenotype displayed by some *Klebsiella* strains, most frequently possessing capsular types KP_K1, KP_K2 or KP_K57. *E. coli* 26561 possesses capsule locus almost indistinguishable from *Klebsiella* K10 (KP_K10) which has not to date been associated with hypermucoviscosity. In *Klebsiella*, this phenotype has also been correlated with capsule-enhancing regulators RmpA and RmpA2 (Lai et al., 2003; Cheng et al., 2010), neither of which is present in 26561 chromosome or either of its two plasmids. This hypermucoviscosity also differs from the enhanced mucoidy observed in some *E. coli* capsule (colanic acid) regulatory mutants. Reports of *E. coli* with mucoviscous phenotype are exceptional with only two previous clinical reports of uropathogenic isolates although these isolates were not characterized in detail (Bottone, 2010; Benham et al., 2013). In the absence of detailed information for those strains, we postulate that the hypermucoviscous phenotype of *E. coli* 26561 is conferred by determinants distinct from those required for this characteristic in *Klebsiella* and from other hypermucoid or mucoviscous phenotypes in *E. coli*.

As noted above, 26561 exhibits mucoviscous phenotype under a broad range of growth conditions thus it is presumed that this phenotype is not subject to measurable regulatory control under the conditions examined. This is consistent with constitutive expression of G1C reported by others. Although not reported in *E. coli*, G1C expression in *Klebsiella* is subject to some regulatory control by iron via Fur (Lin et al., 2011) and IscR (Wu et al., 2014) although neither iron levels nor other environmental variations affected production as assessed by viscosity assay. Whether *E. coli* G1C is subject to additional regulatory inputs and whether this varies from distinct G1C in *E. coli* or the equivalent capsule in *Klebsiella* requires further investigation.

Taking together the preceding observations on polysaccharides and their production in *E. coli* 26561 represent an ideal opportunity for comparative investigation of determinants of the mucoviscous genotype and phenotype.

### O89m Distribution, Isolation Source, and Multidrug Resistance (MDR)

Expanding from the prototypic O89m *E. coli* strain 26561 to an extensive genomic survey identified that *E. coli* serotyped *in silico* as O89 are more frequently O89m than conventional O89 serotype. Extensive population genomic analysis of O89m/O89 strains will be undertaken and reported separately. Only O89m strains also possessed G1C whereas O89 strains possessed colanic acid, consistent with the mutual exclusivity between these polysaccharide loci. As a group, O89m strains tended to lack conventional virulence factors.

Appraisal of available metadata (summarized in **Supplementary Data Sheet 1**) indicated that a substantial number of O89m strains have been isolated from invasive infections (including urinary tract, pleural cavity, bloodstream, mastitis, wound) or as antibiotic-resistant strains thus circumstantially aligning these O89m:G1C strains to ExPEC pathotype. Comprehensive categorization of acquired and mutational resistances among O89 and O89m strains showed a dearth of resistances among the former and a high preponderance of resistance among the latter. Specifically, the O89 strains typically carried zero or one resistance (although two O89 strains can be categorized as multidrug resistant) whilst 89% of the O89m strains can be categorized as MDR with these strains carrying resistance to an average of six antibiotic classes. Carriage of this magnitude of resistances resembles the accumulation of resistances in ST131 strains (Pitout and DeVinney, 2017) although it is unusual among the entirety of *E. coli* (Goldstone and Smith, 2017).

The World Health Organization has identified ESBL- and carbapenemase-producing *E. coli* (and other Enterobacteriaceae) as high priority organisms hence we assessed presence of these resistances in genome content. Profiling this characteristic showed a similarly high density of ESBL, carbapenemase or both among O89m strains. Additionally, approximately 1 in 8 (24 of 206) O89m strains carried the recently emerged *mcr-1* gene (Liu et al., 2016), indicative of resistance to colistin; one additional strain carried a *pmrB* allele (Quesada et al., 2015; Cannatelli et al., 2017) associated with colistin resistance. Two-way plots of features summarized in **Supplementary Data Sheet 1** are provided in **Supplementary Figure 1**. Taken together, these plot permutations re-affirm the preponderance of O89m strains compared with conventional O89 strains and the wide geographical and host species distribution of O89m strains. These plots also indicate an expansion of O89m from the early 2000s concurrent with (i) acquisition of G1C; (ii) accumulation of multiple antibiotic resistances by mutation and horizontal transfer; and (iii) increasing isolation from extra-intestinal sites. Antibiotic resistances gained include ESBLs and carbapenemases. In addition, 10% of O89m isolates carry a *mcr* gene conferring resistance to colistin.

Resistance to antibiotics such as third generation cephalosporins, carbapenems and colistin – considered as “front-line” or “last resort” treatments – identifies O89m as a possessing “pool” of priority resistances. Co-occurrence in O89m strains of these high concern resistances with fitness and virulence determinants such as CPSs in combination with the increasing association of O89m strains with extra-intestinal infections strongly suggest that these *E. coli* strains should be considered as an emergent high risk clade requiring continued monitoring.

## Materials and Methods

### Bacterial Culture and MIC Determination

Bacterial isolates were stored at -80°C in 20% v/v glycerol and were revived by plating onto LB agar and growing overnight at 37°C in air. For each experiment, fresh working cultures were prepared from frozen stocks. A range of solid and liquid microbiological media were used including LB, MH, TSB, BHI, NB, PCA, M9, and MacConkey. For some purposes, *E. coli* were cultured in DMEM defined medium sourced from either Sigma-Aldrich (DMEM-S; catalog number D6046) or Thermo Fisher (DMEM-T; catalog number 11960). Routinely, growth was carried out at a temperature of 37°C and additionally at 24°C and room temperature (c. 18°C) for assessment of mucoidy. For anaerobic growth, culture was carried out using AnaeroGen (Oxoid) or in an A35 Anaerobic Workstation (Don Whitley Scientific, Shipley, United Kingdom) with a gas mixture of hydrogen:carbon dioxide:nitrogen of 10:10:80. Where required, iron availability was adjusted through supplementation with FeSO_4_ (20, 40, 60, 80 or 100 μM final concentration) or incorporation of dipyridyl (10, 20, 50, 100, 200 μM).

### Genome Sequencing, Assembly, and Annotation

Short-reads Assembly: DNA was extracted from an overnight culture *of Escherichia coli* 26561 using MasterPure^TM^ DNA Purification Kit from Epicentre. Sequencing was performed by Glasgow Polyomics using the MiSeq platform. Raw data was assessed with FastQC^1^. Reads were pre-processed using MyPro (Liao et al., 2015) prior to assembly using SPAdes 3.1.1 (Bankevich et al., 2012). The quality of the assembly was evaluated using Quast (Gurevich et al., 2013). The assembled genome consisted of 96 contigs (NG50 141, 083) with an average coverage ×15 and total length of 4.76 Mb.

Long-reads Assembly: DNA was extracted from a separate culture of strain 26561 and sequenced using the PacBio platform. Pbh5tools from Pacific Biosciences was installed locally and bashh5tools used to extract subreads from the bax files in fastq format. These reads were used in combination with the raw sequence data from the MiSeq platform mentioned above [which was this time pre-processed using ngsShoRT (Chen et al., 2014)] to produce a hybrid assembly using SPAdes 3.9.1.

Assemblies were annotated using Prokka 1.12 (Seemann, 2014) and visualized in ARTEMIS. Coding sequences in regions of interest were manually checked using BLAST and corrected where appropriate. Visualizations of alignments between 26561 and other genomes were produced using ARTEMIS (Rutherford et al., 2000) and EasyFig 2.2 (Sullivan et al., 2011).

*Escherichia coli* 26561 was previously sequenced in short reads format and deposited at NCBI (BioSample SAMN04334740; GenBank assembly accession GCA_001575995.1. Assemblies generated from PaBio sequencing have been deposited as BioProject SAMN08202527 with GenBank accession numbers CP027118.1 (chromosome), CP027119.1 (86,731 bp plasmid) and CP027120.1 (54,399 bp plasmid).

### *Escherichia coli* Genomes and Genotypic Characterization

This was carried out using a panel of webtools accessed via Center for Genomic Epidemiology. Specifically, the tools used were SerotypeFinder (Joensen et al., 2015), MLST typer (Larsen et al., 2012), ResFinder (Zankari et al., 2012), and PlasmidFinder (Carattoli et al., 2014). The panel of *E. coli* O89 genomes listed in **Supplementary Data Sheet 1** were sourced from NCBI.

### Protein Sample Preparation and Mass Spectrometry

A fresh colony of 26561 was used to inoculate 5 ml of DMEM (Sigma D6046) and incubated overnight at 37°C in anaerobic conditions. From this point, to minimize fluctuations in conditions, inoculation, incubation, centrifugation, and lysis were all conducted in the same tube used for culture of each replicate: In brief, 12 high-speed centrifuge tubes (Oak Ridge PSF) were filled with 30 ml of warmed DMEM. Four were supplemented with Ceftazidime (Sigma, C0690500, Batch 2, 200 mg. Eur.Pharma. Reference standard) to a concentration of 0.15 μg/ml and another 4 to a concentration of 0.3 μg/ml (respectively, approximately ¼ and ½ MIC as determined under experimental conditions used, i.e., DMEM under anaerobic conditions).

Each was inoculated with 30 μl of the overnight culture (1/1000 dilution). The cultures were incubated anaerobically at 37°C for 6 h after which they were transferred immediately to a pre-warmed (37°C) high-speed centrifuge (Sigma 4K15) set at 13,500 rpm for 14 min (spin conditions established in prior tests to determine minimum time for formation of pellets). The pellets obtained were drained of media, and 2 ml lysis solution (100 mM Tris-HCL, pH 7.6 containing 4% SDS) was added. Samples were incubated in lysis buffer for 10 min at 95°C, vortexed, and then flash frozen for immediate transfer to the proteomics facility on dry ice. Prior to freezing, aliquots were retained for assay of protein yield using Bio-Rad RC-DC (detergent-compatible) kit. Yields were equivalent to approximately 1 mg/ml lysate and were measured also by the proteomics facility.

### Proteomic Analysis – TMT Labeling and High pH Reversed-Phase Chromatography

Aliquots of 100 μg of 10 samples per experiment were digested with trypsin (2.5 μg trypsin per 100 μg protein; 37°C, overnight), labeled with TMT10plex reagents according to the manufacturer’s protocol (Thermo Fisher Scientific, Loughborough, United Kingdom) and the labeled samples pooled.

An aliquot of 50 μg of the pooled sample was evaporated to dryness and resuspended in buffer A (20 mM ammonium hydroxide, pH 10) prior to fractionation by high pH reversed-phase chromatography using an Ultimate 3000 liquid chromatography system (Thermo Fisher Scientific). In brief, the sample was loaded onto an XBridge BEH C18 Column (130 Å, 3.5 μm, 2.1 mm × 150 mm, Waters, United Kingdom) in buffer A and peptides eluted with an increasing gradient of buffer B (20 mM Ammonium Hydroxide in acetonitrile, pH 10) from 0 to 95% over 60 min. The resulting fractions were evaporated to dryness and resuspended in 1% formic acid prior to analysis by nano-LC MSMS using an Orbitrap Fusion Tribrid mass spectrometer (Thermo Scientific).

### Nano-LC Mass Spectrometry

High pH RP fractions (total proteome analysis) or phospho-enriched fractions (phospho-proteome analysis) were further fractionated using an Ultimate 3000 nanoHPLC system in line with an Orbitrap Fusion Tribrid mass spectrometer (Thermo Scientific). In brief, peptides in 1% (vol/vol) formic acid were injected onto an Acclaim PepMap C18 nano-trap column (Thermo Scientific). After washing with 0.5% (vol/vol) acetonitrile 0.1% (vol/vol) formic acid peptides were resolved on a 250 mm × 75 μm Acclaim PepMap C18 reverse phase analytical column (Thermo Scientific) over a 150-min organic gradient, using seven gradient segments (1–6% solvent B over 1 min, 6–15% B over 58 min, 15–32% B over 58 min, 32–40% B over 5 min, 40–90% B over 1 min, held at 90% B for 6 min and then reduced to 1% B over 1 min) with a flow rate of 300 nl min^-1^. Solvent A was 0.1% formic acid and Solvent B was aqueous 80% acetonitrile in 0.1% formic acid. Peptides were ionized by nano-electrospray ionization at 2.0 kV using a stainless-steel emitter with an internal diameter of 30 μm (Thermo Scientific) and a capillary temperature of 275°C.

All spectra were acquired using an Orbitrap Fusion Tribrid mass spectrometer controlled by Xcalibur 2.0 software (Thermo Scientific) and operated in data-dependent acquisition mode using an SPS-MS3 workflow. FTMS1 spectra were collected at a resolution of 120,000, with an automatic gain control (AGC) target of 200,000 and a max injection time of 50 ms. The most intense ions were selected for MS/MS. Precursors were filtered according to charge state (to include charge states 2–7) and with monoisotopic precursor selection. Previously interrogated precursors were excluded using a dynamic window (40 s ± 10 ppm). The MS2 precursors were isolated with a quadrupole mass filter set to a width of 1.2 m/z. ITMS2 spectra were collected with an AGC target of 5000, max injection time of 120 ms and CID collision energy of 35%.

For FTMS3 analysis, the Orbitrap was operated at 60,000 resolution with an AGC target of 50,000 and a max injection time of 120 ms. Precursors were fragmented by high energy collision dissociation (HCD) at a normalized collision energy of 55% to ensure maximal TMT reporter ion yield. Synchronous precursor selection (SPS) was enabled to include up to five MS2 fragment ions in the FTMS3 scan.

### Protein Identification and Quantification

MaxQuant 1.5.7.4 (Tyanova et al., 2016a) was used to identify proteins from the raw MS files. The 4577 protein sequences from our 26561 hybrid assembly, together with the contaminant sequences provided by MQ, were used to form the search database. Modifications were selected as ‘fixed’ for Carbamidomethyl and ‘variable’ for Oxidation (M) and Acetyl (Protein N-term). Phospho (STY) was added as ‘variable’ for the files related to phospho-enriched samples. The reporter ion was MS3, TMT10plex, with a reporter mass tolerance of 0.01 D. The digestion mode was trypsin with two missed cleavages permitted. The PSM and Protein FDR was 1%. Only peptides mapping uniquely to one protein sequence were used for quantification. Data was further processed and analyzed using Perseus 1.5.5.0 (Tyanova et al., 2016b). Data are available via ProteomeXchange with identifier PXD008339 and a summary of the relevant peptides and protein identification data is provided as **Supplementary Data Sheet 2**.

### Viscosity Assay

This utilized a simple centrifugation-based assay to quantify bacterial sedimentation at low centrifugal force (Lai et al., 2003; Cheng et al., 2010). Briefly, standardized inocula were prepared in medium and cultured overnight (18–20 h). Total growth was monitored by measuring absorbance at a wavelength of 600 nm (A_600_); aliquots were placed into 1.5 ml microcentrifuge tubes and centrifuged (1,000 ×*g*; 4°C; 5 min). Tubes were carefully handled to minimize mixing and the upper 1 ml of liquid was gently aspirated into a pipette tip and dispensed into microcuvettes for A_600_ measurement. The ratio of A_600_ for post-centrifuged (lower value) to the pre-centrifuged (greater value) was calculated to estimate relative viscosity. Relative viscosity values of <0.2 were categorized as “negative”; LOW values as 0.2–0.35; MEDIUM values as 0.5–0.8, and HIGH values as >0.8.

### Cellular and Extra-Cellular Polysaccharide Preparation

*E. coli* for LPS and CPS preps were cultured overnight at 37°C. The LPS and CPS were extracted as described previously by Hitchcock and Brown (1983), with modifications of Geisinger and Isberg (2015) to reduce nucleic acids content in the sample preps. Cellular polysaccharides were extracted from cell pellets and extracellular polysaccharides from supernatants after centrifugation at 15,000 ×*g* for 10 min (which is required to sufficiently sediment 26561). Silver staining was performed using Bio-Rad silver stain kit (catalog number 1610443), according to manufacturer’s instructions. Alcian blue staining (8GX, Alfa Aesar, Fisher Scientific, catalog number 15432949) was performed according to methodology described by Mercaldi et al. (2008).

## Author Contributions

SH, FR, and DS conceived and designed the work. SH, MP, RQ, FR, and DS generated, analyzed, and interpreted the data. SH and DS composed the manuscript. All authors contributed to further appraisal and editing of the work and approved the content for submission.

## Conflict of Interest Statement

FR is an employee of DC Biosciences Ltd. which provided proteomics capabilities and initial data management for this work. Their participation involved an “in-kind” contribution. The remaining authors declare that the research was conducted in the absence of any commercial or financial relationships that could be construed as a potential conflict of interest.

## Footnotes

1 http://www.bioinformatics.babraham.ac.uk/projects/fastqc
