# Supplementary material for: Variant O89 O-Antigen of E. coli Is Associated With Group 1 Capsule Loci and Multidrug Resistance
Source: Front Microbiol. 2018 Aug 31;9:2026. doi: 10.3389/fmicb.2018.02026 (PMC6128206; doi:10.3389/fmicb.2018.02026)
Supplement: Supplementary file 1 [file Table_1.docx]

Supplementary Material

**Variant O89 O-antigen of *E. coli* is associated with Group 1 Capsule loci and multidrug resistance**

**Susan Harris, Marta J Piotrowska, Robert J Goldstone, Ruby (Dan) Qi, Geoffrey Foster, Ulrich Dobrindt, Jean-Yves Madec, Charlotte Valat, Francesco V Rao, David G E Smith^*^**

*** Correspondence:**

David G E Smith, Institute of Biological Chemistry, Biophysics & Bioengineering, Heriot-Watt University, Edinburgh, EH14 4AS, UK
David.smith@hw.ac.uk

Supplementary Table 1(A) : annotation of genes in group I capsule (G1C) locus of *E. coli* 26561

Supplementary Table 1(B) : 26561 O-antigen gene annotation

Supplementary Table 1(C) : Presence of remnant O8/O9 O-antigen locus among E. coli O89m strains

Supplementary Table 1(D): *E. coli* O89m strains with intact genomic region spanning *ybdK–nfsB*

**SUPPLEMENTARY Table 1A** – **annotation of genes in group I capsule (G1C) locus of *E. coli* 26561**

| **26561 gene no.** | ***Gene name*** | **Annotation** | [Sequence](https://www.ncbi.nlm.nih.gov/protein/24266667?report=genbank&log$=protalign&blast_rank=2&RID=RYGHWBYZ01R) **match*** |
| --- | --- | --- | --- |
| 1663 | *galF* | UDP-glucose pyrophosphorylase | [AAN52283.1](https://www.ncbi.nlm.nih.gov/protein/24266667?report=genbank&log$=protalign&blast_rank=2&RID=RYGHWBYZ01R) |
| 1664 |  | phosphatase PAP2 family protein | [WP_059336834.1](https://www.ncbi.nlm.nih.gov/protein/979512213?report=genbank&log$=protalign&blast_rank=1&RID=RYGHWBYZ01R) |
| 1665 | *insB* | Transposase | [WP_000951585.1](https://www.ncbi.nlm.nih.gov/protein/446874329?report=genbank&log$=protalign&blast_rank=1&RID=RYGHWBYZ01R) |
| 1666 | *wzi* | capsule assembly Wzi family protein | [WP_059336855.1](https://www.ncbi.nlm.nih.gov/protein/979512272?report=genbank&log$=protalign&blast_rank=1&RID=RYGHWBYZ01R) |
| 1667 | *wza* | polysaccharide export protein | [WP_059336856.1](https://www.ncbi.nlm.nih.gov/protein/979512274?report=genbank&log$=protalign&blast_rank=1&RID=RYGHWBYZ01R) |
| 1668 | *wzb* | protein tyrosine phosphatase | [WP_059336857.1](https://www.ncbi.nlm.nih.gov/protein/979512277?report=genbank&log$=protalign&blast_rank=1&RID=RYGHWBYZ01R) |
| 1669 | *wzc* | tyrosine protein kinase | [WP_061362250.1](https://www.ncbi.nlm.nih.gov/protein/1001954794?report=genbank&log$=protalign&blast_rank=1&RID=RYGHWBYZ01R) |
| 1670 | *wbaP* | undecaprenyl-phosphate galactose phosphotransferase | [WP_059336859.1](https://www.ncbi.nlm.nih.gov/protein/979512282?report=genbank&log$=protalign&blast_rank=1&RID=RYGHWBYZ01R) |
| 1671 |  | hypothetical (glycosyl-transferase) | [WP_059336860.1](https://www.ncbi.nlm.nih.gov/protein/979512286?report=genbank&log$=protalign&blast_rank=1&RID=RYGHWBYZ01R) |
| 1672 |  | hypothetical (glycosyl-transferase) | [WP_059336861.1](https://www.ncbi.nlm.nih.gov/protein/979512289?report=genbank&log$=protalign&blast_rank=1&RID=RYGHWBYZ01R) |
| 1673 |  | hypothetical | [WP_077875601.1](https://www.ncbi.nlm.nih.gov/protein/1149948083?report=genbank&log$=protalign&blast_rank=1&RID=RYGHWBYZ01R) |
| 1674 |  | glycosyl transferase family 1 protein | [WP_049110280.1](https://www.ncbi.nlm.nih.gov/protein/896064936?report=genbank&log$=protalign&blast_rank=1&RID=RYGHWBYZ01R) |
| 1675 |  | glycosyl transferase family 1 protein | [WP_059336864.1](https://www.ncbi.nlm.nih.gov/protein/979512295?report=genbank&log$=protalign&blast_rank=1&RID=RYGHWBYZ01R) |
| 1676 |  | hypothetical (glycosyl hydrolase family 1) | [WP_059336865.1](https://www.ncbi.nlm.nih.gov/protein/979512298?report=genbank&log$=protalign&blast_rank=1&RID=RYGHWBYZ01R) |
| 1677 |  | O-antigen ligase domain-containing protein | [WP_077875600.1](https://www.ncbi.nlm.nih.gov/protein/1149948082?report=genbank&log$=protalign&blast_rank=1&RID=RYGHWBYZ01R) |
| 1678 |  | hypothetical protein (acetyl transferase) | [WP_072310665.1](https://www.ncbi.nlm.nih.gov/protein/1119333456?report=genbank&log$=protalign&blast_rank=1&RID=RYGHWBYZ01R) |
| 1679 | *wbaZ* | glycosyl transferase family 4 protein | [WP_059336700.1](https://www.ncbi.nlm.nih.gov/protein/979511860?report=genbank&log$=protalign&blast_rank=1&RID=RYGHWBYZ01R) |
| 1680 | *gnd* | NADP-dependent 6-phosphogluconate dehydrogenase | [WP_059336698.1](https://www.ncbi.nlm.nih.gov/protein/979511857?report=genbank&log$=protalign&blast_rank=1&RID=RYGHWBYZ01R) |
| 1681 | *cpsB/manC* | mannose-1-phosphate guanylyltransferase/  mannose-6-phosphate isomerase | [WP_059336696.1](https://www.ncbi.nlm.nih.gov/protein/979511854?report=genbank&log$=protalign&blast_rank=1&RID=RYGHWBYZ01R) |
| 1682 | *cpsG/manB* | phosphomannomutase | [WP_000192835.1](https://www.ncbi.nlm.nih.gov/protein/446114980?report=genbank&log$=protalign&blast_rank=1&RID=RYGHWBYZ01R) |
| 1683 | *Ugd* | UDP-glucose 6-dehydrogenase | [WP_000704906.1](https://www.ncbi.nlm.nih.gov/protein/446627560?report=genbank&log$=protalign&blast_rank=1&RID=RYGHWBYZ01R) |

*** - sequence matches were 100% identical over 100% of the sequence length**

**SUPPLEMENTARY Table 1B – 26561 O-antigen gene annotation**

| **26561 gene no.** | **Gene name** | **Annotation** | **Match length** | **length** | **% ID (blastp)** | **%coverage** |
| --- | --- | --- | --- | --- | --- | --- |
| 3140 | *wcaM* | colanic acid biosynthesis protein wcaM domain protein | 263 | 462 | 94 | 56 |
| 3139 | *galF* | GalU regulator GalF | 297 | 297 | 99 | 100 |
| 3138 | *gale* | UDP-glucose 4-epimerase GalE | 338 | 338 | 95 | 100 |
| 3137 |  | glycosyl transferase family 1 | 752 | 738 | 95 | 100 |
| 3136 | *Wzm* | O-antigen ABC transporter permease subunit Wzm | 259 | 259 | 99 | 100 |
| 3135 | *Wzt* | O-antigen ABC transporter ATP-binding protein Wzt | 250 | 250 | 98 | 100 |
| 3134 |  | glycosyltransferase, group 1 family protein | 343 | 343 | 91 | 100 |
| 3133 |  | glycosyltransferase, group 1 family protein | 374 | 373 | 91 | 97 |
| 3132 |  | class I SAM-dependent methyltransferase | 390 | 380 | 47 | 98 |
| 3131 |  | glycosyl transferase family 1 | 681 | 658 | 42 | 99 |
| 3130 | *Gnd* | 6-phosphogluconate dehydrogenase | 33 | 468 | 96 | 4 |

**SUPPLEMENTARY TABLE 1C – Presence of remnant O8/O9 O-antigen locus among E. coli O89m strains**

Remnants of O8/O9 loci in *E. coli* O89m strains are adjacent to group I capsule locus between *ugd* and *hisI*. Table 3 details the BLASTn matches to the region between *ugd* *and his* from the strains which are illustrated in the main text in Figure 6. Matches to *Klebsiella* capsule obtained using the Kaptive webtool are presented.

| Strain | Kaptive result | # CDS remnants | BLASTn query length | Aligns to serotype | % ID (query coverage = 100%) |
| --- | --- | --- | --- | --- | --- |
| 26561 | K10 | 1 (incomplete) | 195 | O8 or O9* | 100 |
| Y5 | K115 | 1 (incomplete) | 194 | O8 or O9* | 100 |
| Sanji | K30 | 1 (incomplete) | 825 | O9 | 99 |
| Ec590 | K53 | 1 (incomplete) | 825 | O9 | 100 |
| H8 | K31 | 3 | 3126 | O8 | 99 |
| 6409 | K127 | 6 | 9129 | O9 | 99 |

# * Reference sequences for *mtfC*, the final gene of the O8 and O9 loci, are identical over the 3’-terminal 228 nucleotides.

# SUPPLEMENTARY TABLE 1D – *E. coli* O89m strains with intact genomic region spanning *ybdK–nfsB*.

Assemblies for the following O89m strains were downloaded and annotated. Each was submitted to Kaptive for capsule typing which was confirmed manually.

Ec_B41 SAMN02436334

Ec_G188 SAMN04334716

Ec_G229 SAMN04334733

Ec_G233 SAMN04334734

Ec_G315 SAMN04334753

Ec_LSBS01 SAMN04423153

The genes conforming ‘O89m’ were located and adjacent genes upstream and downstream sought and identified where feasible.

In all except G315, O8/O9 remnants were located immediately upstream to *his* operon. In G315, it was not possible to locate O89m as both *ugd* and *hisI* were located at contig ends.

In each case other than ecB41 (which is composed of only 5 contigs), these genes formed small contigs on their own, presumably due to the presence of insertion sequences at either end, hence a location could not be determined. The *ybdK-ybdF-ybdJ-nfsB* genes were confirmed as being contiguous, and so we can only state that the O89m locus is not situated in this region according to the given assembly.

| Strain | Kaptive K-type | Location of O89m |
| --- | --- | --- |
| ecB41 | KL20 (incomplete) | *acrB-tomB*-**O89m***-dtpD-phr-ybdF* |
| Ec_G188 | KL16 | Unknown – not *ybdK-nfsB* |
| Ec_G229 | KL127 | Unknown – not *ybdK-nfsB* |
| Ec_G233 | KL55 | Unknown – not *ybdk-nfsB*, or *tomB-dtpD* region |
| Ec_G315 | KL10 (incomplete) | Unknown – not *ybdK-nfsB* |
| Ec_LSBS01 | KL127 | Unknown – not *ybdK-nfsB*, or *tomB-dtpD* region |

**
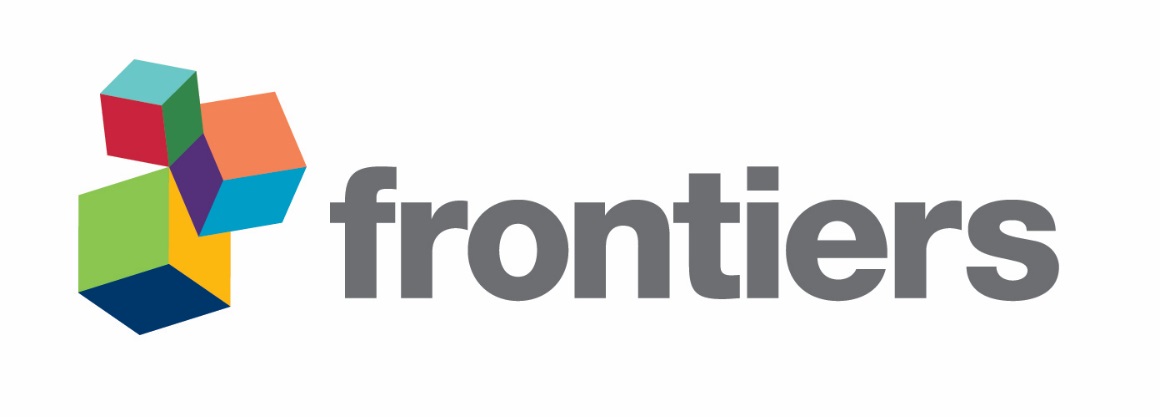
**
