# Supplementary material for: Variant O89 O-Antigen of E. coli Is Associated With Group 1 Capsule Loci and Multidrug Resistance
Source: Front Microbiol. 2018 Aug 31;9:2026. doi: 10.3389/fmicb.2018.02026 (PMC6128206; doi:10.3389/fmicb.2018.02026)
Supplement: Supplementary file 2 [file Presentation_1.PPTX]

## Slide 1
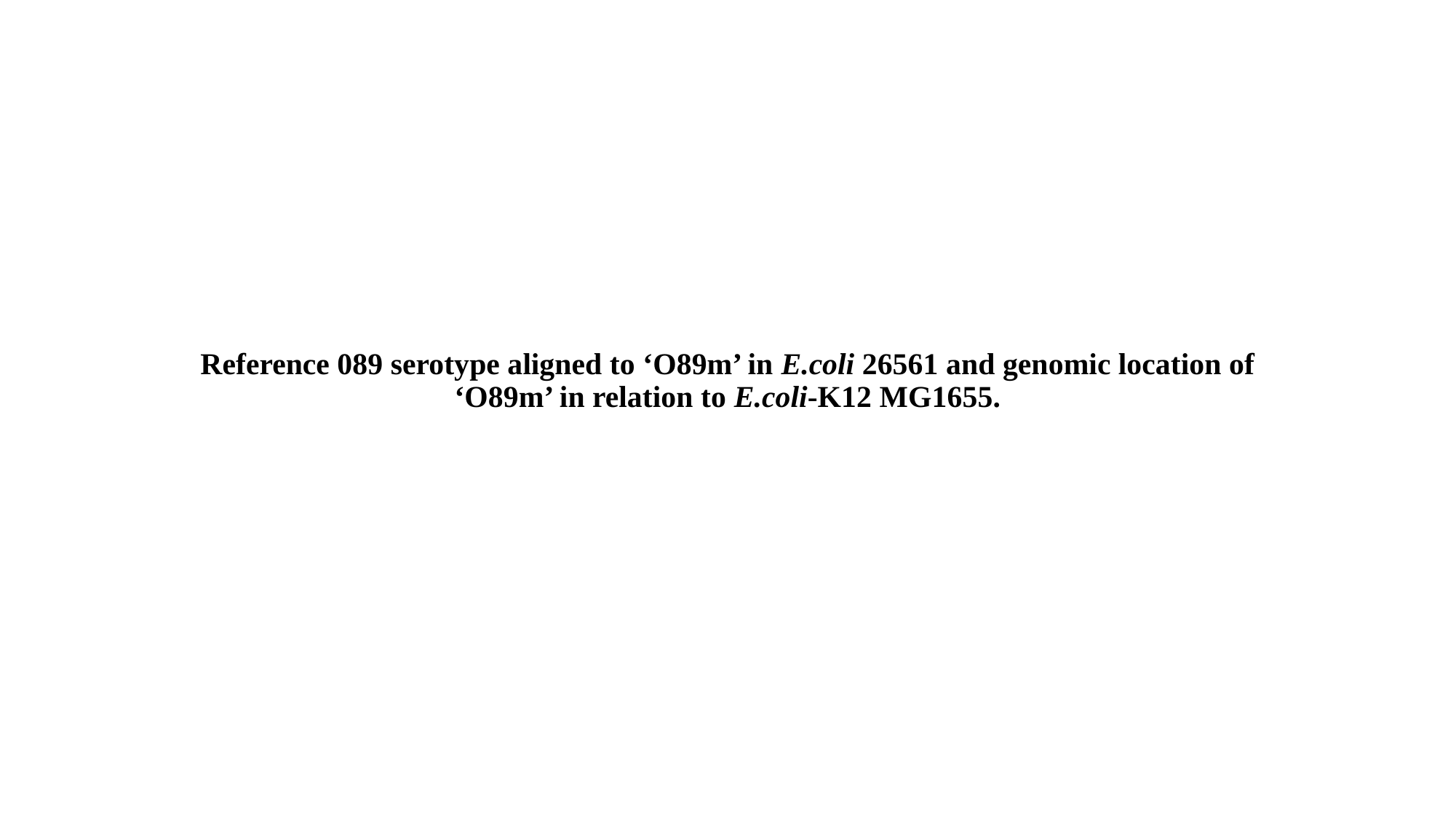

# Reference 089 serotype aligned to ‘O89m’ in E.coli 26561 and genomic location of ‘O89m’ in relation to E.coli-K12 MG1655.

## Slide 2
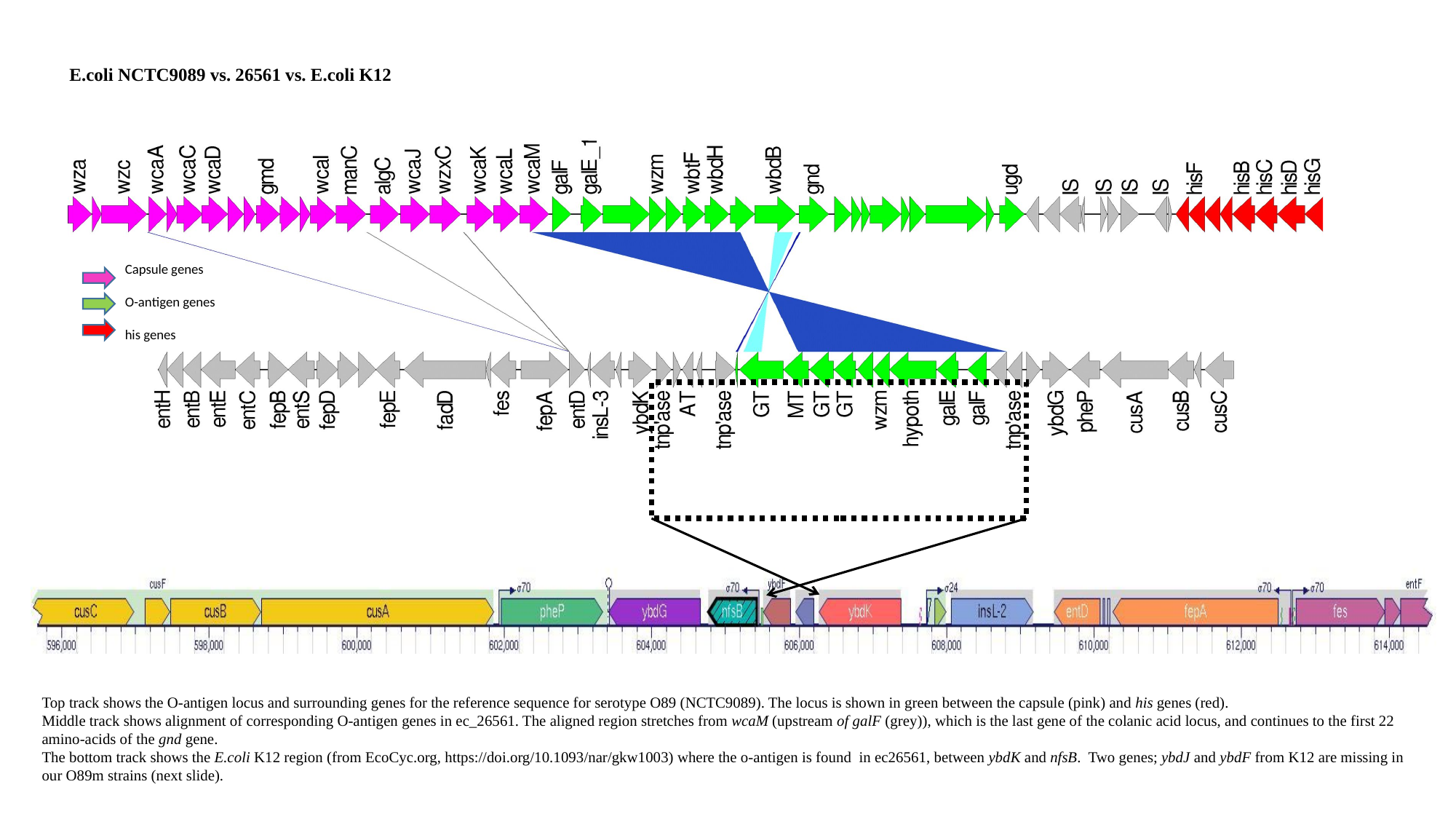

# E.coli NCTC9089 vs. 26561 vs. E.coli K12
Capsule genes
O-antigen genes
his genes
Top track shows the O-antigen locus and surrounding genes for the reference sequence for serotype O89 (NCTC9089). The locus is shown in green between the capsule (pink) and his genes (red).
Middle track shows alignment of corresponding O-antigen genes in ec_26561. The aligned region stretches from wcaM (upstream of galF (grey)), which is the last gene of the colanic acid locus, and continues to the first 22 amino-acids of the gnd gene.
The bottom track shows the E.coli K12 region (from EcoCyc.org, https://doi.org/10.1093/nar/gkw1003) where the o-antigen is found in ec26561, between ybdK and nfsB. Two genes; ybdJ and ybdF from K12 are missing in our O89m strains (next slide).

## Slide 3
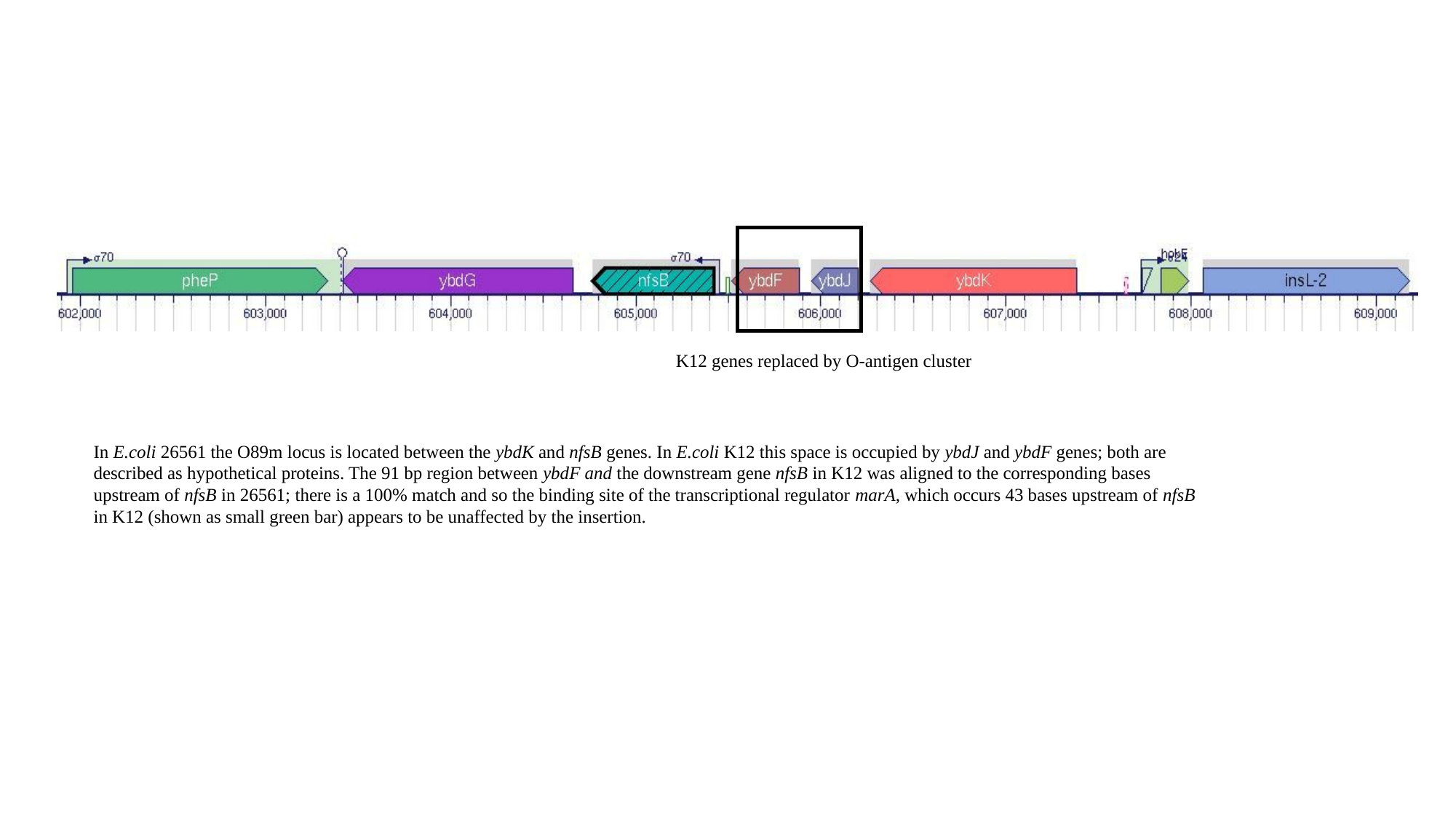

K12 genes replaced by O-antigen cluster
In E.coli 26561 the O89m locus is located between the ybdK and nfsB genes. In E.coli K12 this space is occupied by ybdJ and ybdF genes; both are described as hypothetical proteins. The 91 bp region between ybdF and the downstream gene nfsB in K12 was aligned to the corresponding bases upstream of nfsB in 26561; there is a 100% match and so the binding site of the transcriptional regulator marA, which occurs 43 bases upstream of nfsB in K12 (shown as small green bar) appears to be unaffected by the insertion.
