# Supplementary material for: Variant O89 O-Antigen of E. coli Is Associated With Group 1 Capsule Loci and Multidrug Resistance
Source: Front Microbiol. 2018 Aug 31;9:2026. doi: 10.3389/fmicb.2018.02026 (PMC6128206; doi:10.3389/fmicb.2018.02026)
Supplement: Supplementary file 5 [file Image_1.PDF]

Plots of serotype O89 and O89m strains generated from Supplementary table 2

January 2018

Contents

'O89' Serotype Isolates - Country and Year Isolated 2
'O89' Serotype Isolates - Host vs. Year Isolated 2
Sample Site vs. Year 3
Capsule K-type vs. Year 3
Capsule K-type vs. Host 4
Capsule K-type vs. Sample Site 5
Capsule K-type vs. Country 6
Accessory beta-lactamase (by class) vs. Year 7
Accessory beta_lactamase (all) vs. Year 8
Accessory beta-lactamase (by class) vs. Host 9
Accessory beta-lactamse (by class) vs. K-type 10
Accessory beta-lactamse (by class) vs. Country 11
Mutational Resistances vs. Year 12
Mutational Resistances vs. Host 13
Mutational Resistances vs. Sample Site 14
Mutational Resistances vs. Country 15

The following plots were generated using the 'R' package ggplot2 (H. Wickham. ggplot2: Elegant Graphics for Data Analysis. Springer-Verlag New York, 2009.) Individual points in each plot are 'jittered' to aid visualisation of overlapping points.

The data used to generate the following plots is available in Supplementary table 4, which includes information relating to *E.coli* isolotes which return a match for serotype 089 using SerotypeFinder. For the purposes of easier visualisation, columns were added to the original table to reduce the number of groups within the data while retaining the information in the original. For example, the column 'host_tidy' was added to represent the column 'host_species' where instances of 'BOINE', 'BOVINE' 'CATTLE', 'COW' and 'CALF' are all replaced by 'COW'. Other columns added for visualisation purposes were; 'country_only' (for 'Country'), and 'sample_tidy' (for 'Sample_type').

'O89' Serotype Isolates - Country and Year Isolated

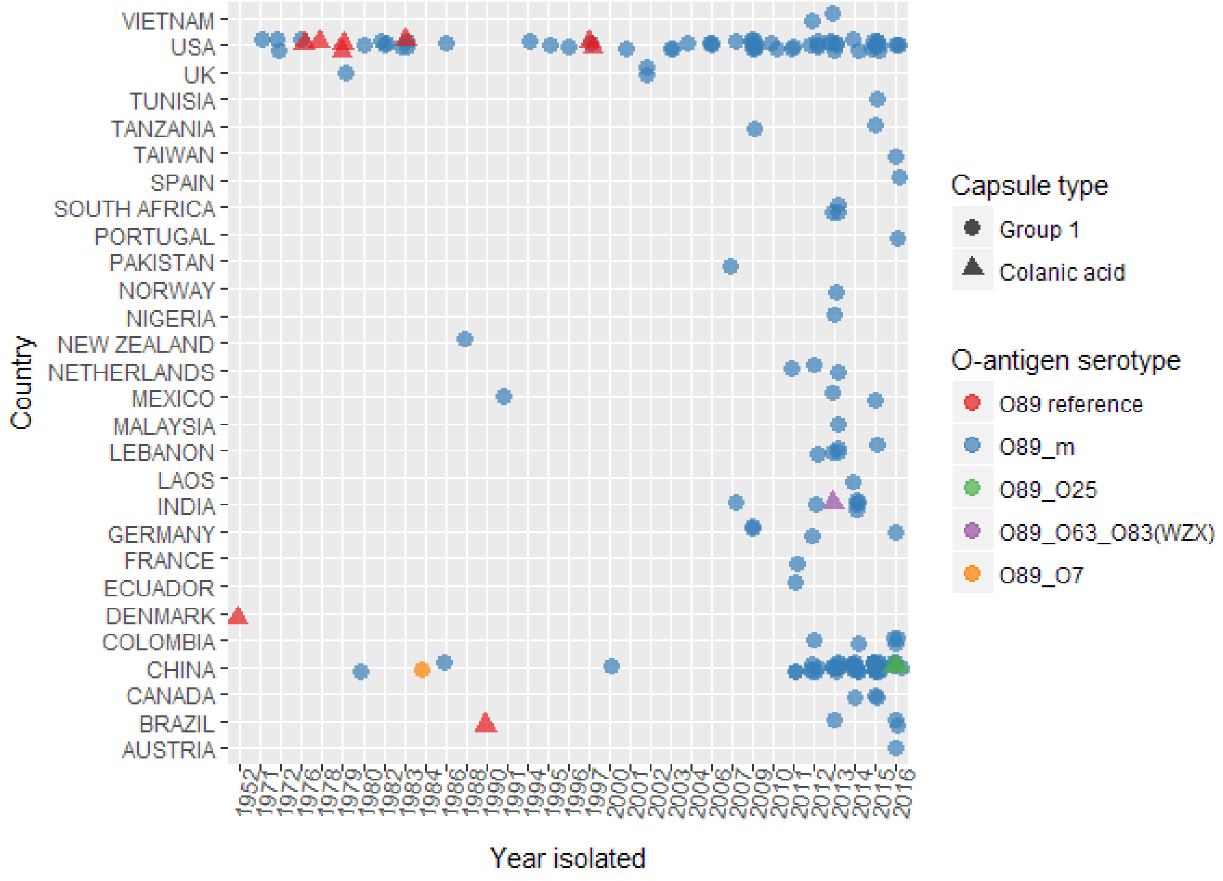

'O89' Serotype Isolates - Host vs. Year Isolated

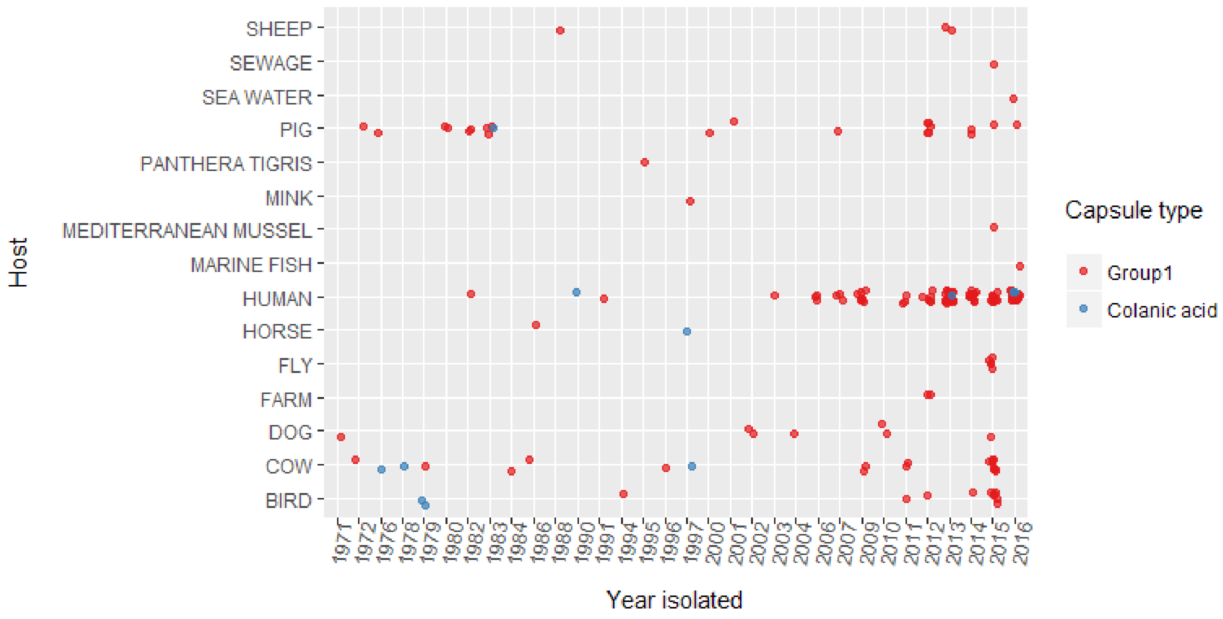

Sample Site vs. Year

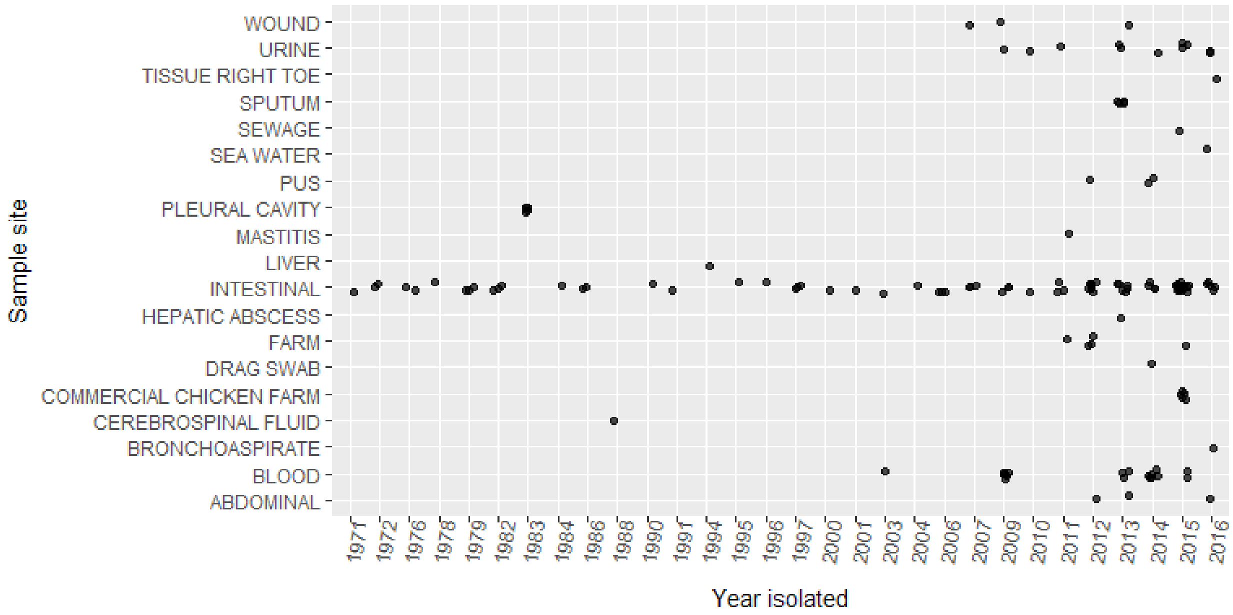

Capsule K-type vs. Year

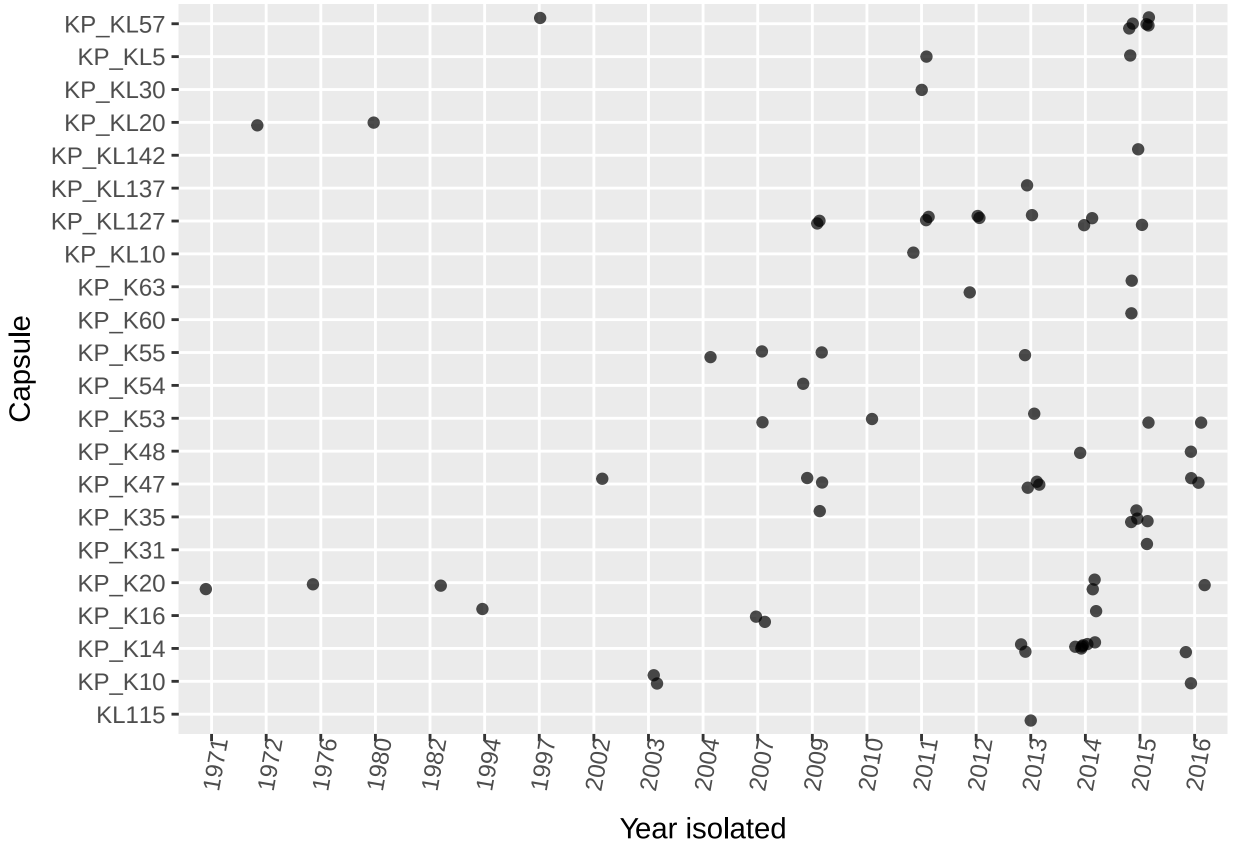

Capsule K-type vs. Host

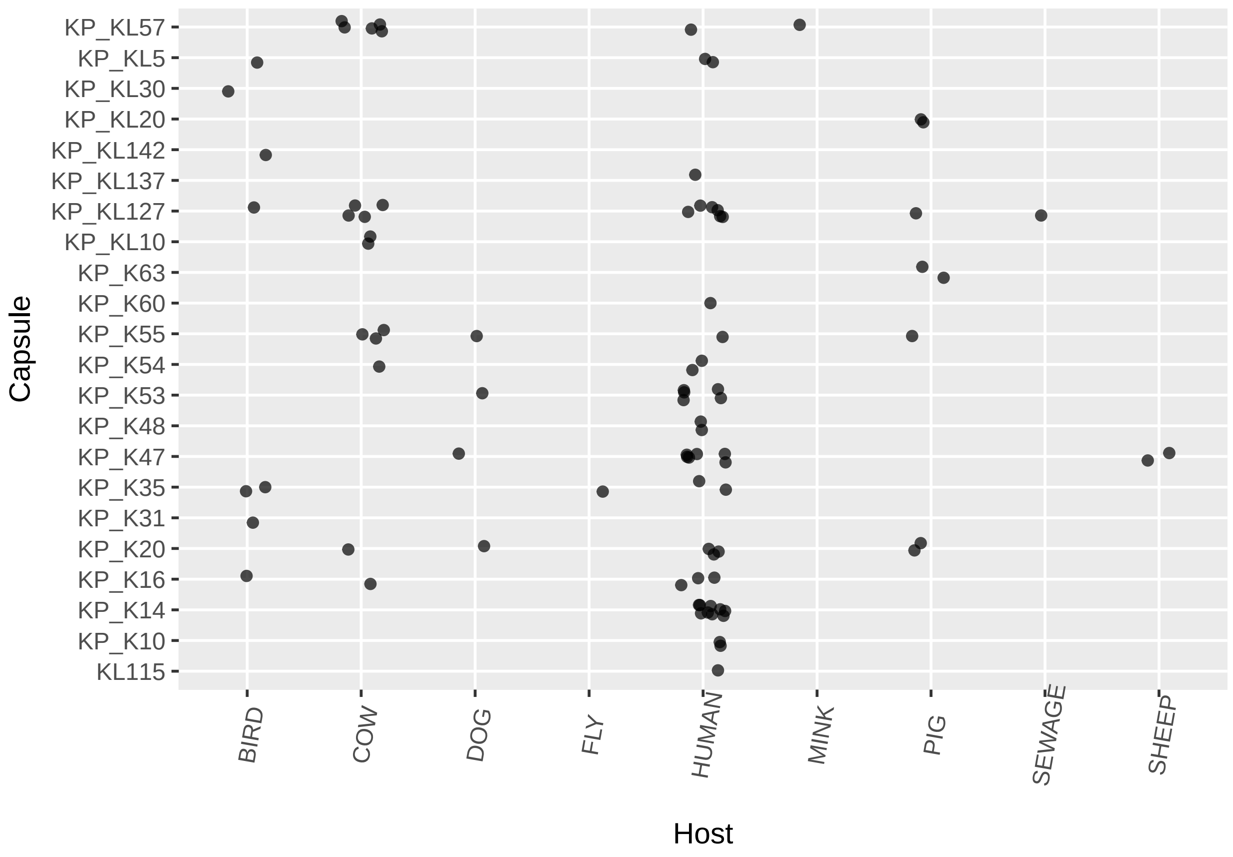

Capsule K-type vs. Sample Site

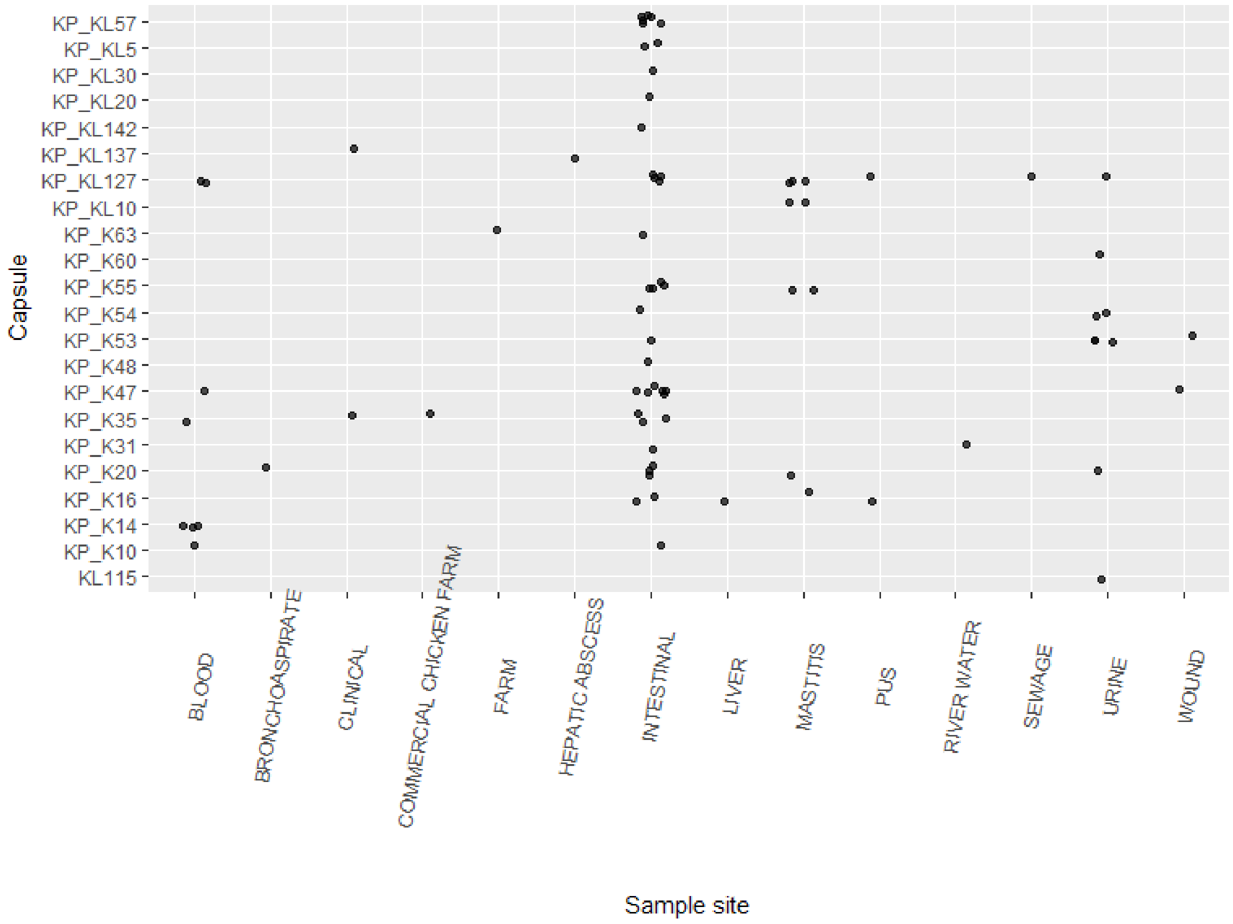

Capsule K-type vs. Country

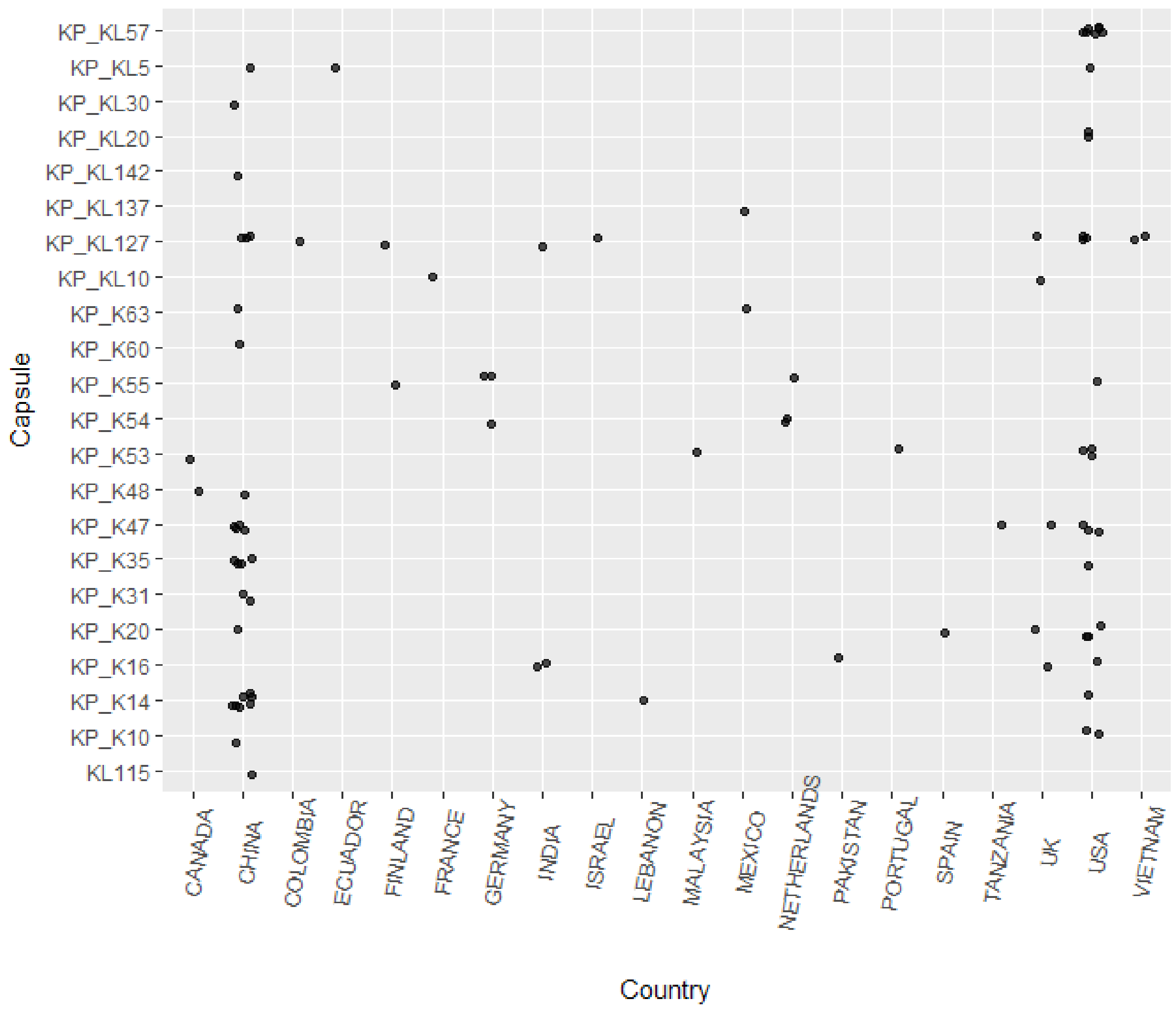

Accessory beta-lactamase (by class) vs. Year

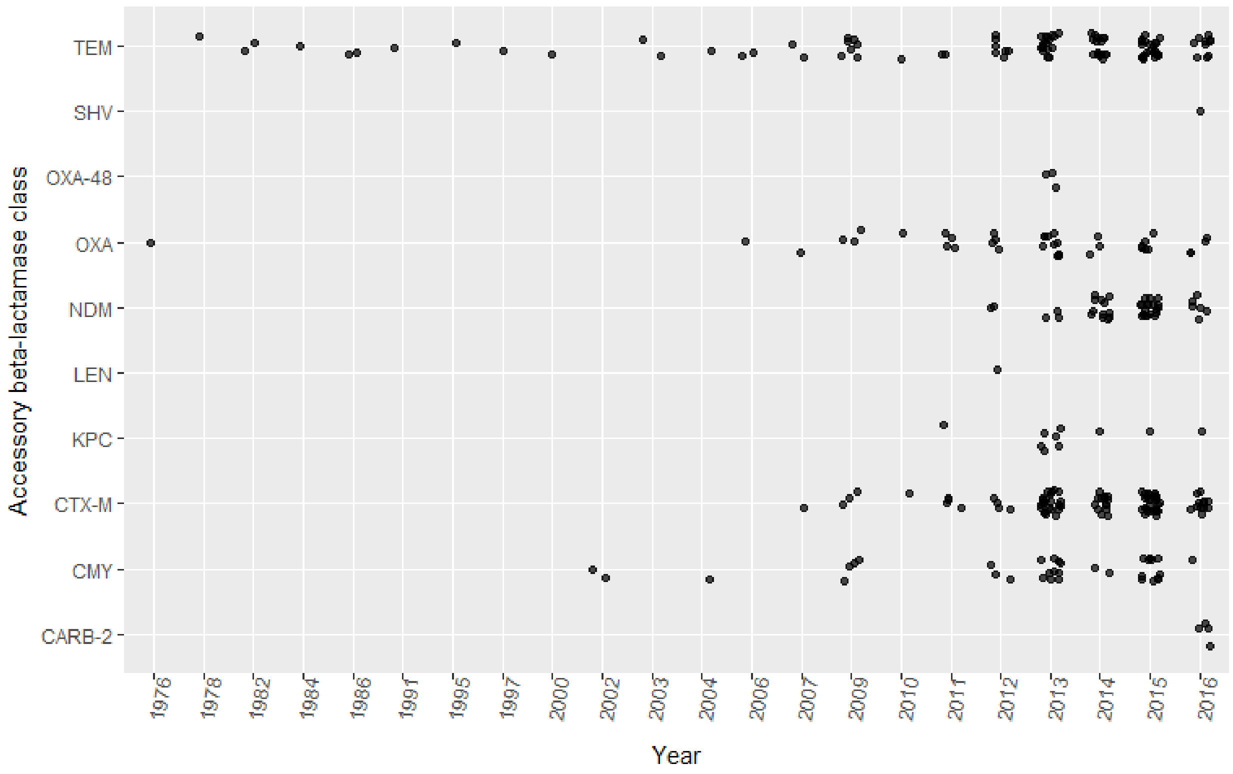

Accessory beta_lactamase (all) vs. Year

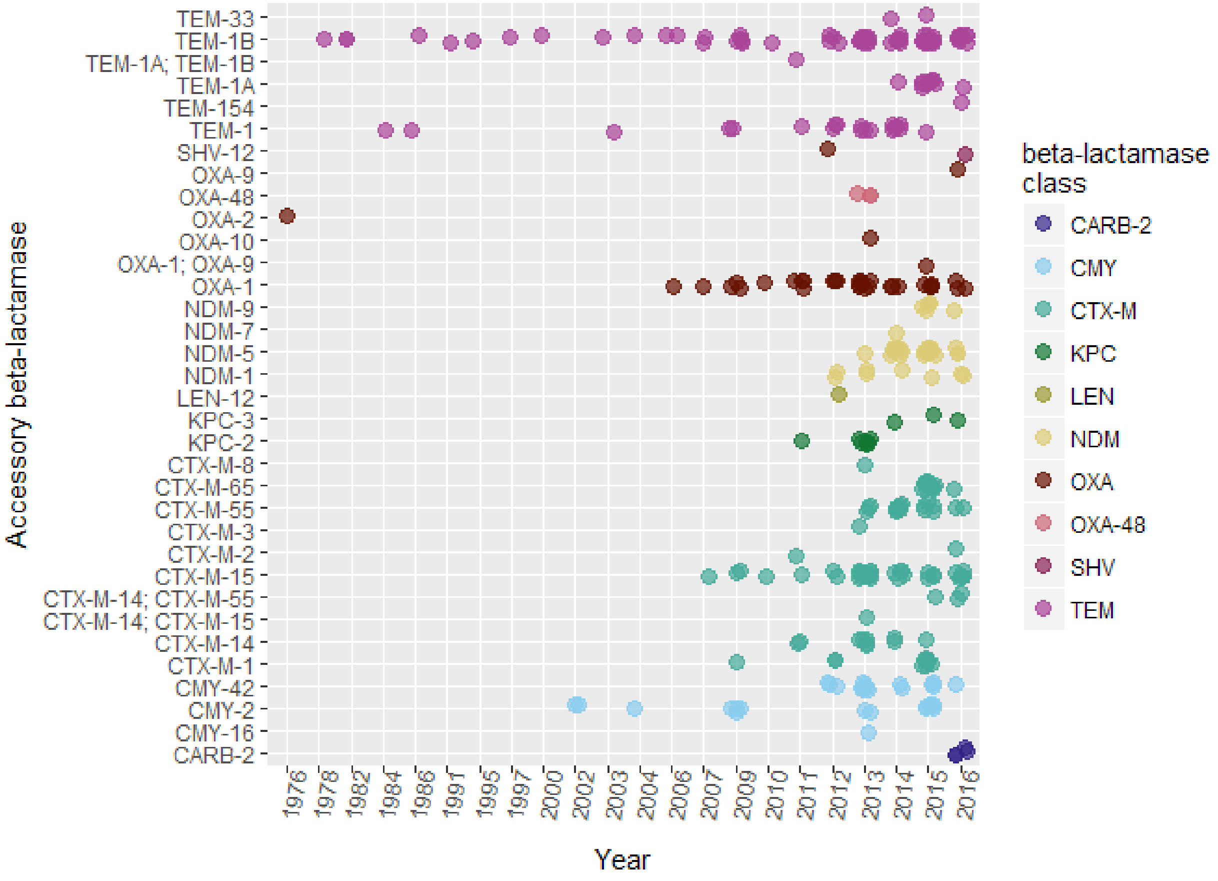

Accessory beta-lactamase (by class) vs. Host

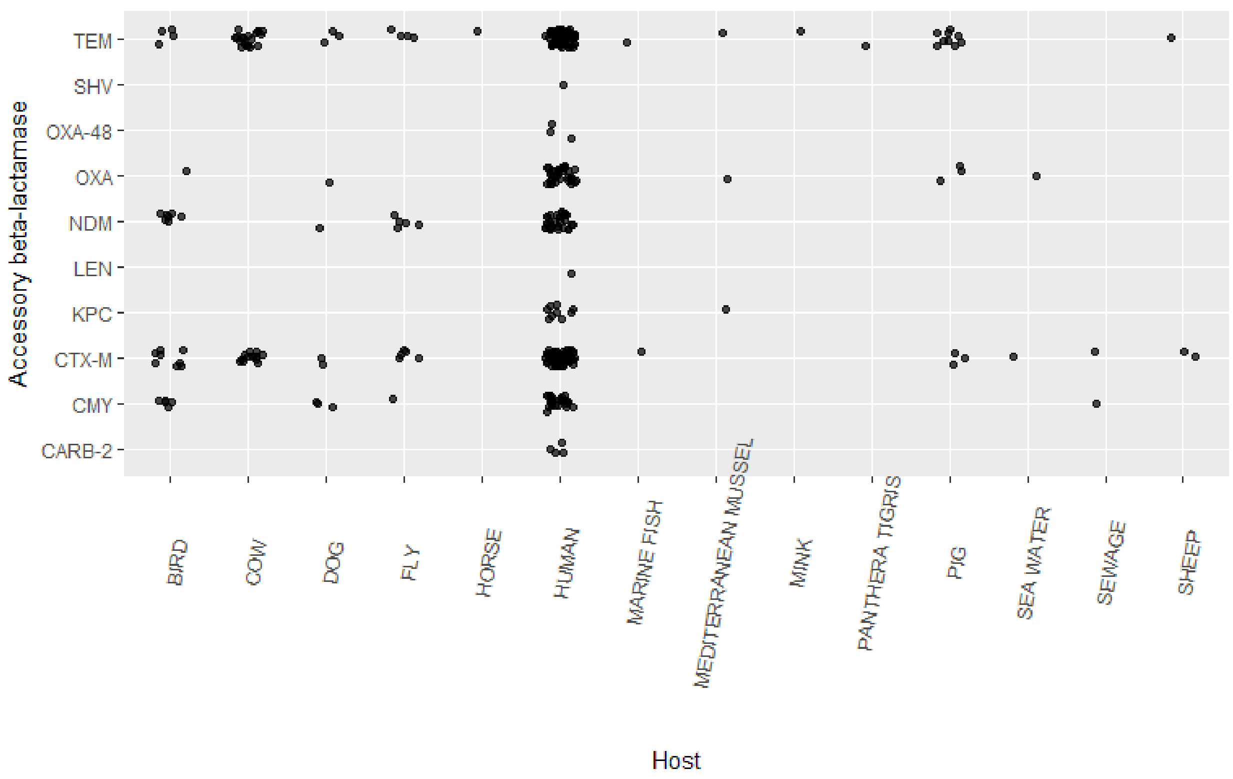

Accessory beta-lactamse (by class) vs. K-type

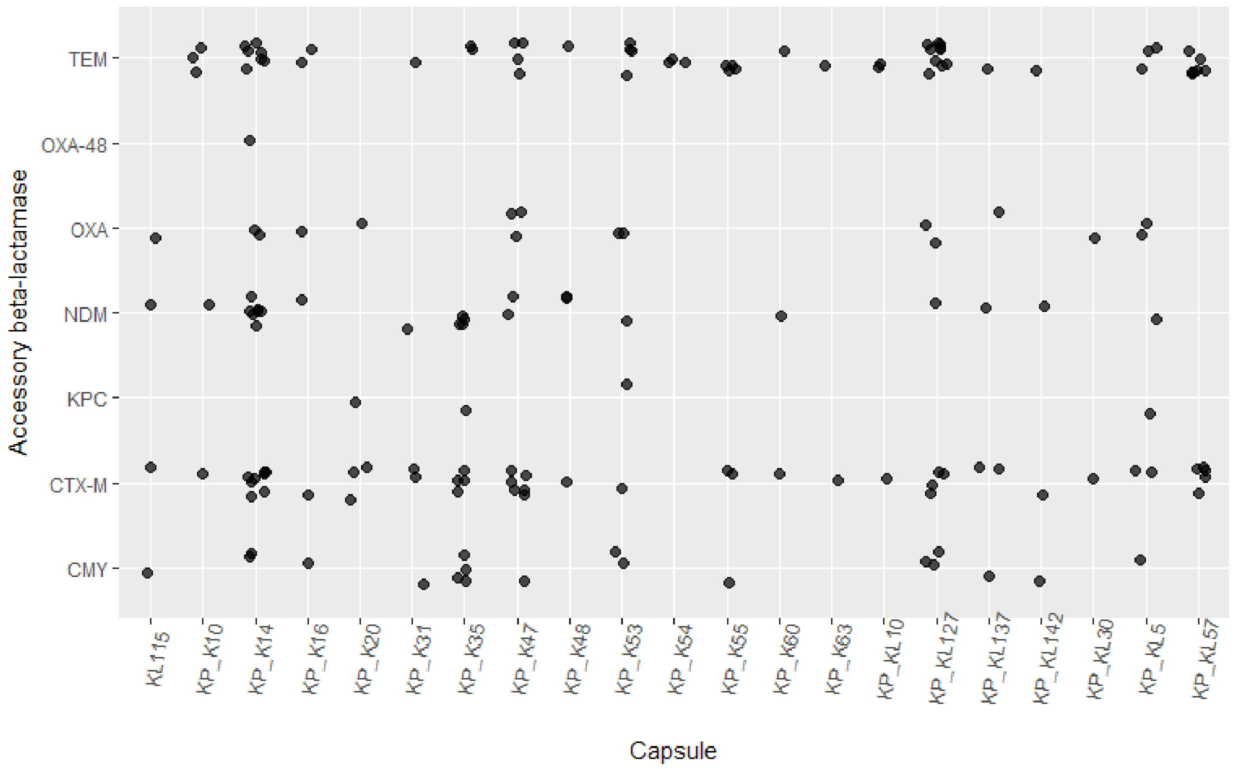

Accessory beta-lactamse (by class) vs. Country

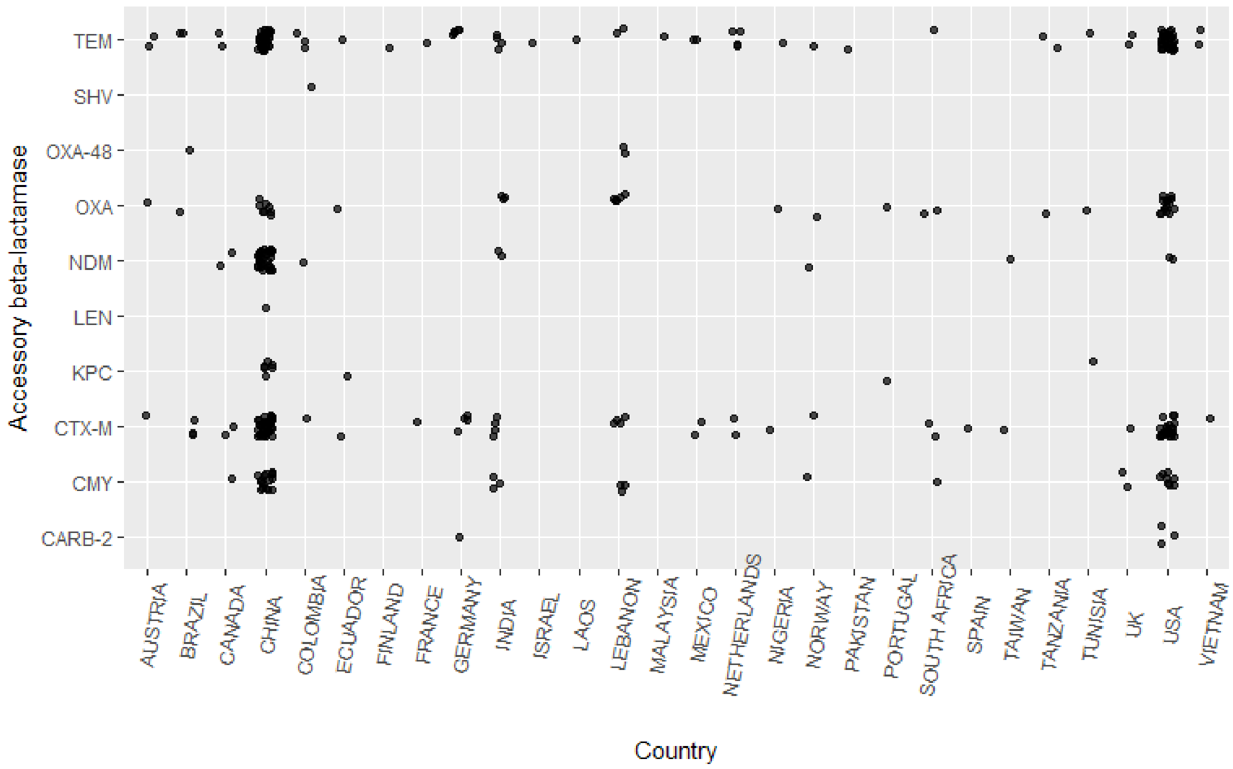

Mutational Resistances vs. Year

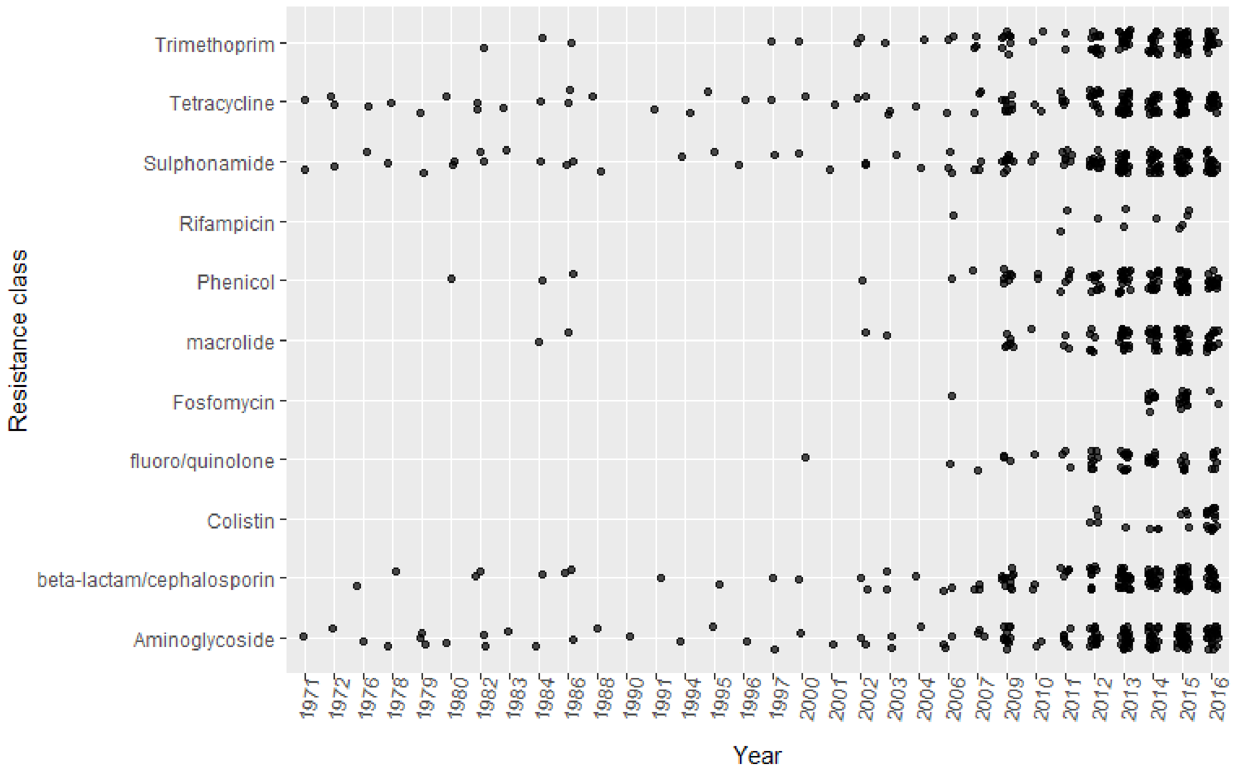

Mutational Resistances vs. Host

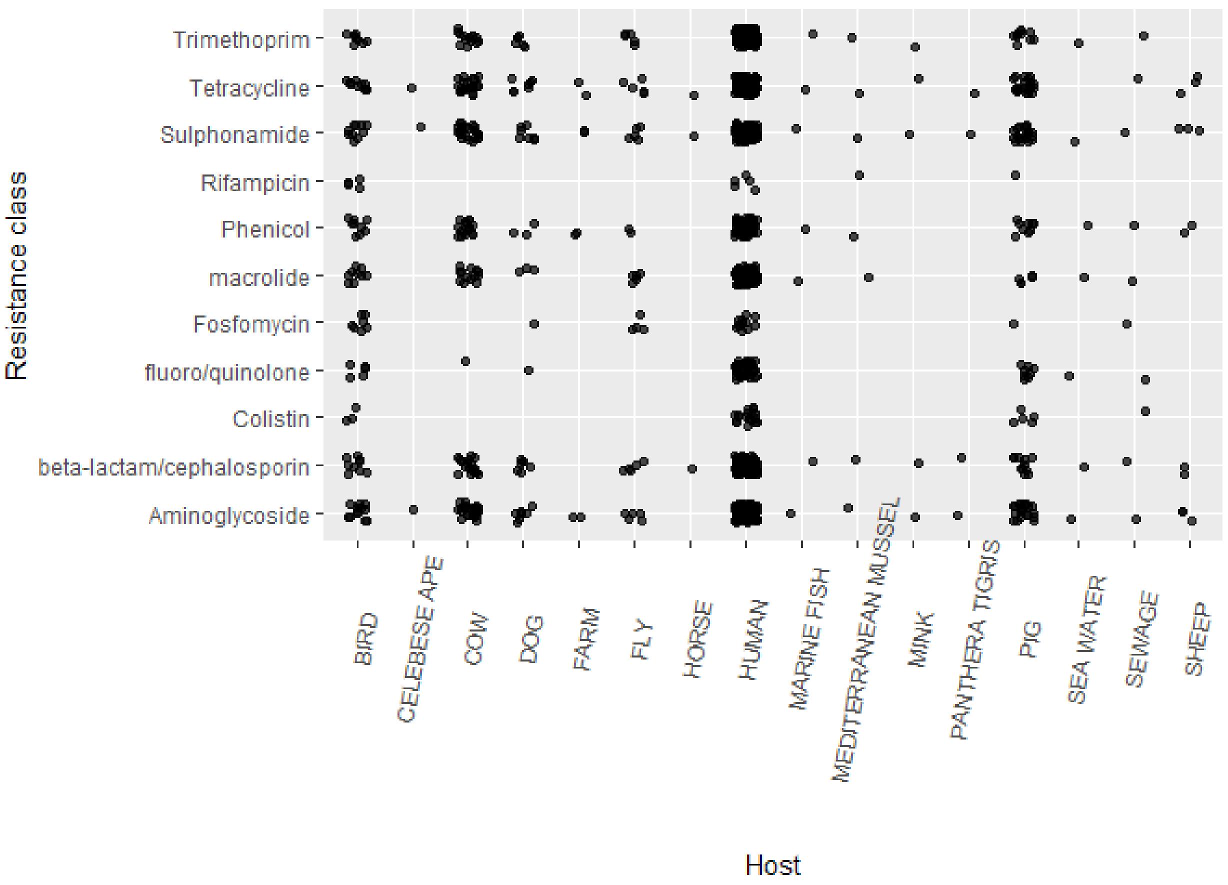

Mutational Resistances vs. Sample Site

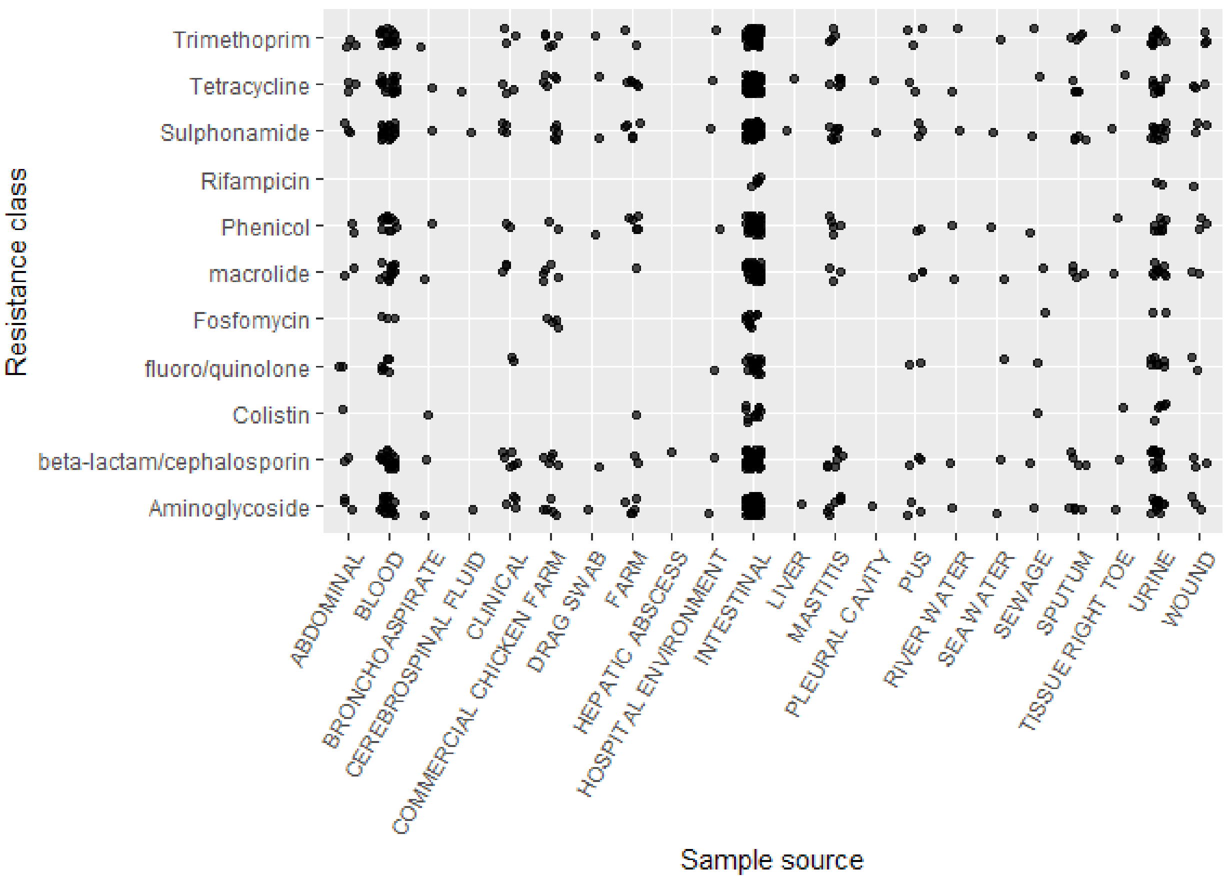

Mutational Resistances vs. Country

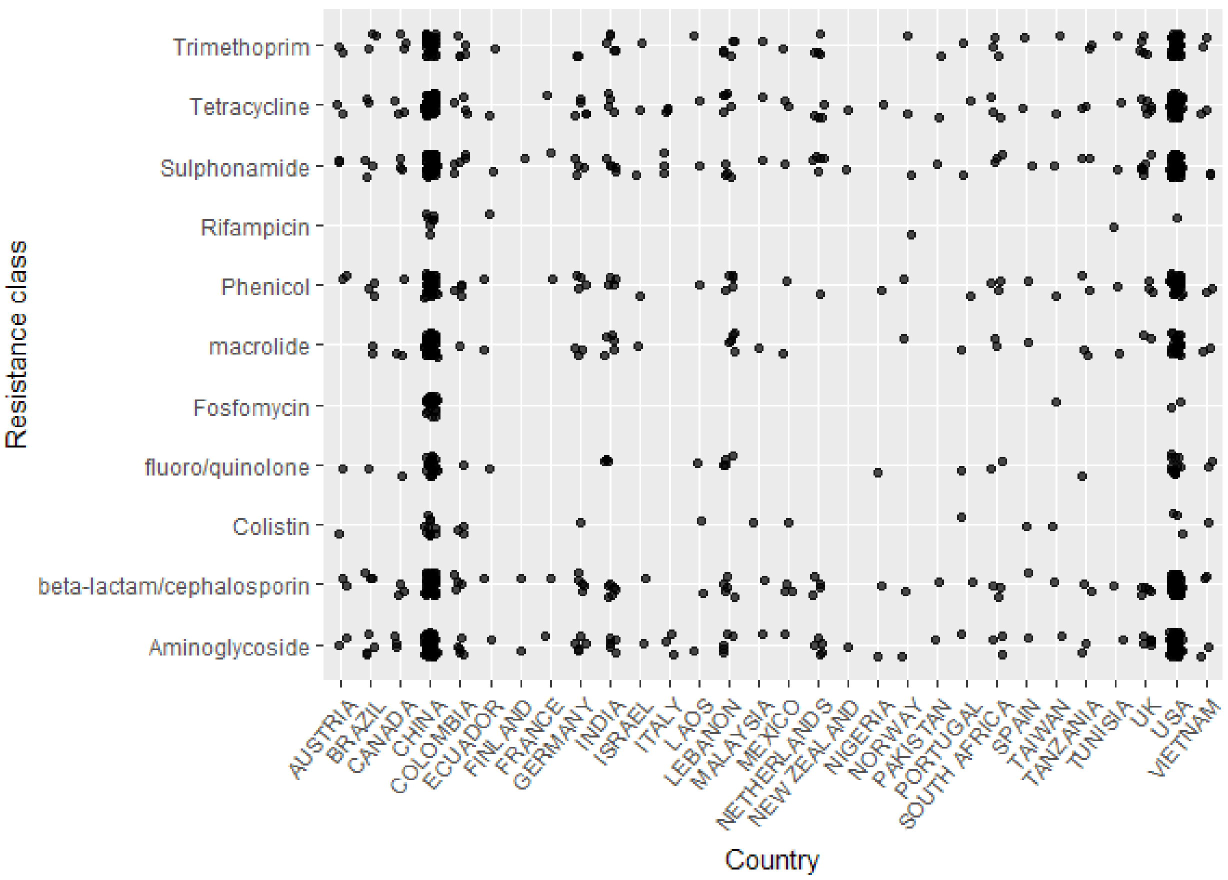

“‘
