# Supplementary material for: Variant O89 O-Antigen of E. coli Is Associated With Group 1 Capsule Loci and Multidrug Resistance
Source: Front Microbiol. 2018 Aug 31;9:2026. doi: 10.3389/fmicb.2018.02026 (PMC6128206; doi:10.3389/fmicb.2018.02026)

ORIGINAL SDS-PAGE GEL IMAGES

A. Silver-stained gel of cell-associated polysaccharide preparation. Presented as Figure 8A

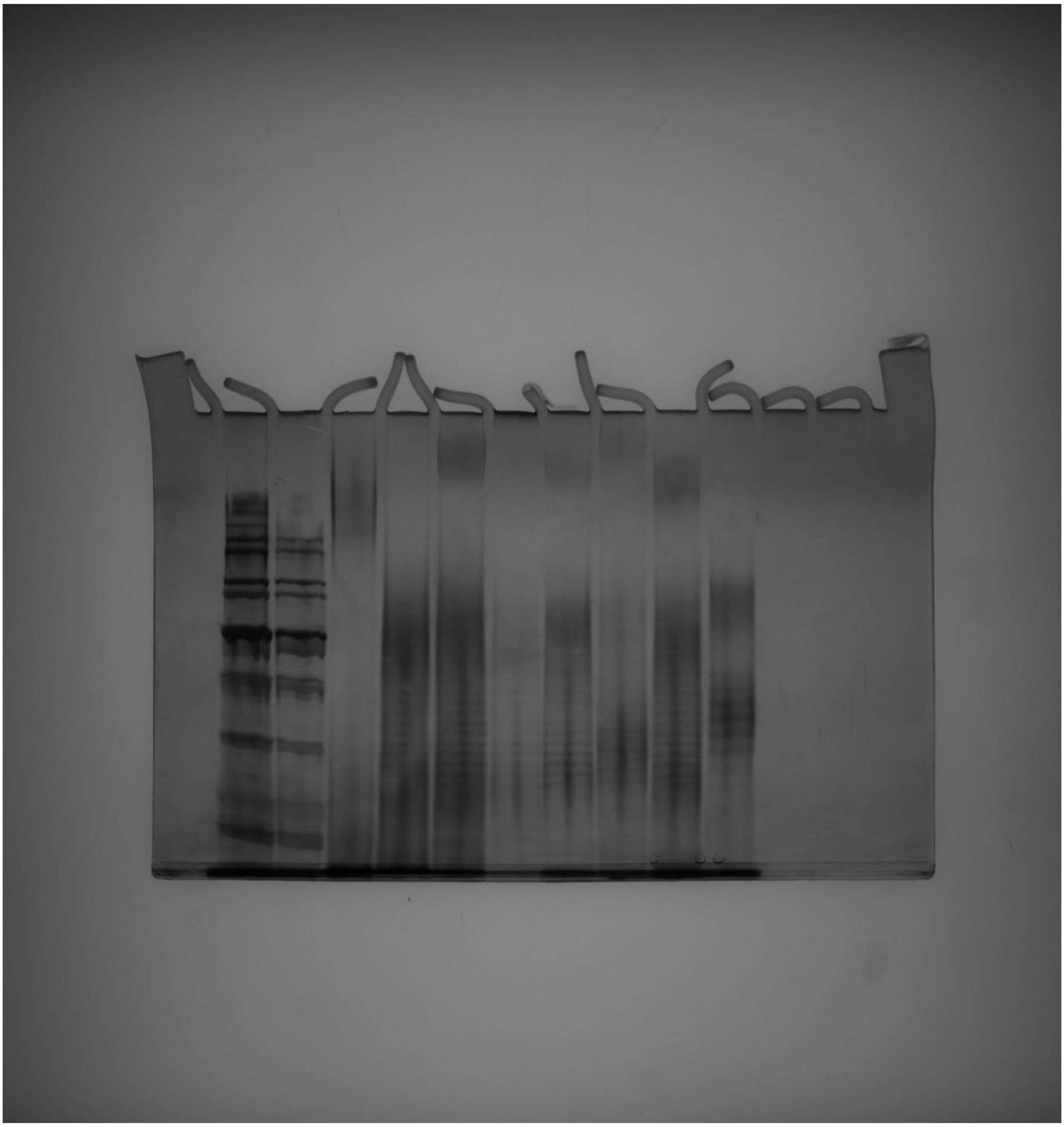

B. Alcian Blue-stained gel of cell-associated polysaccharide preparation. Presented as Figure 8B

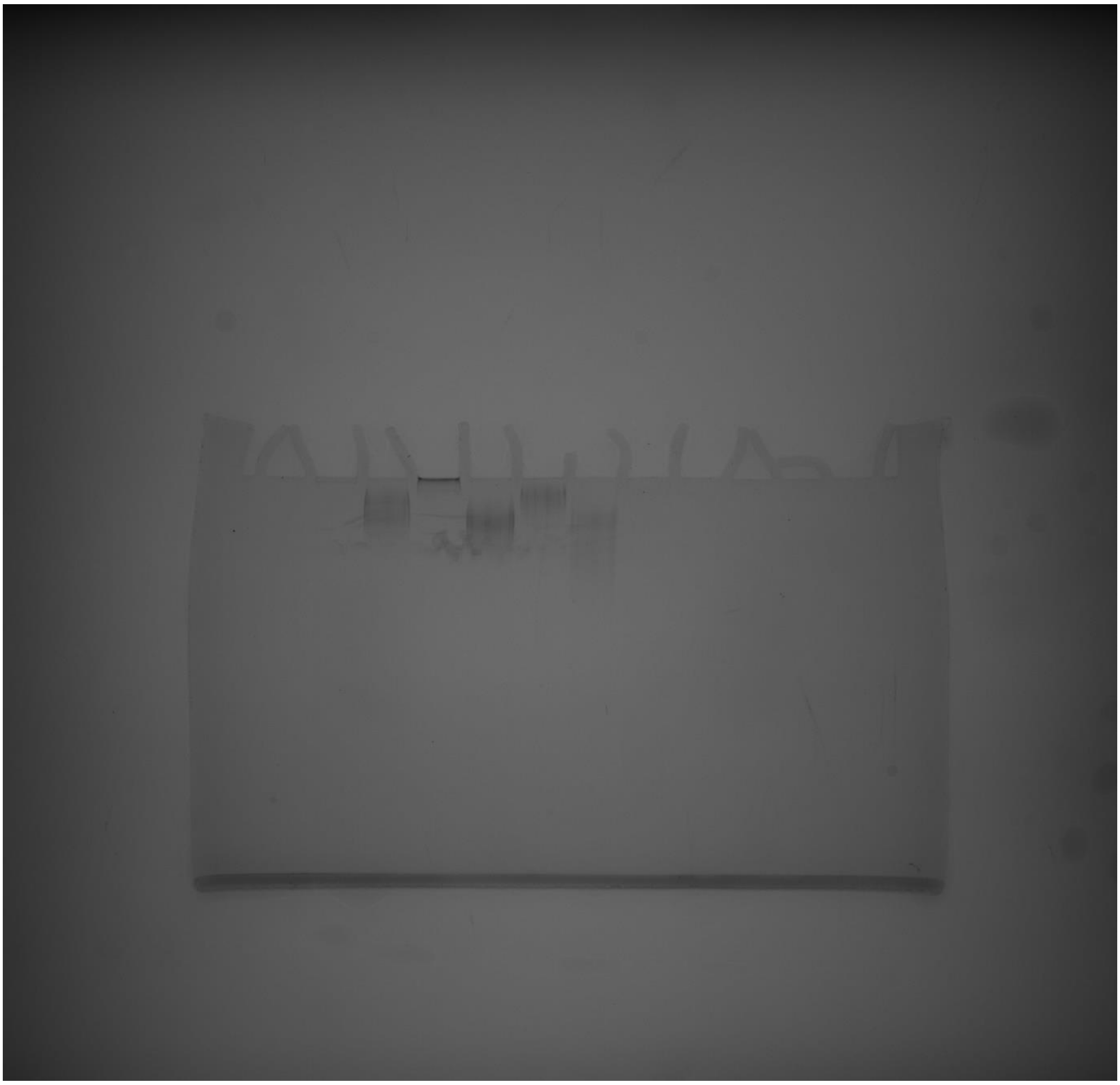

C. Silver-stained gel of extracellular polysaccharide preparation. Presented as Figure 8C

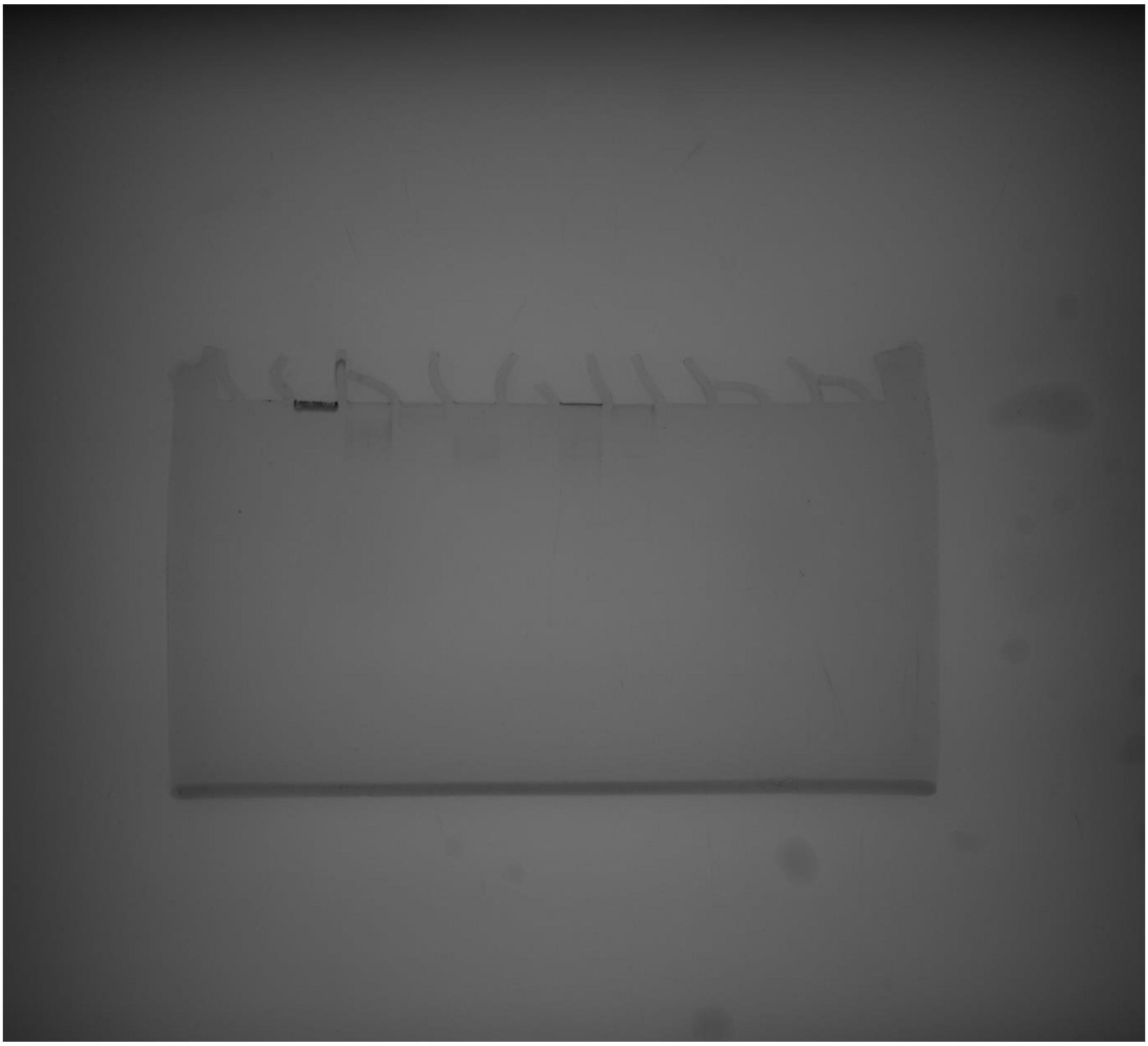

Supplement: Supplementary file 6 [file Image_2.PDF]
